# Identification of PIK3CG as a hub in septic myocardial injury using network pharmacology and weighted gene co‐expression network analysis

**DOI:** 10.1002/btm2.10384

**Published:** 2022-08-03

**Authors:** Qiong Liu, Yushu Dong, Germaine Escames, Xue Wu, Jun Ren, Wenwen Yang, Shaofei Zhang, Yanli Zhu, Ye Tian, Darío Acuña‐Castroviejo, Yang Yang

**Affiliations:** ^1^ Key Laboratory of Resource Biology and Biotechnology in Western China Ministry of Education, Faculty of life Science and Medicine, Northwest University Xi'an China; ^2^ Xi'an Key Laboratory of Cardiovascular and Cerebrovascular Diseases Xi'an No. 3 Hospital, The Affiliated Hospital of Northwest University, Faculty of life Science and Medicine, Northwest University Xi'an China; ^3^ Institute of Neuroscience, General Hospital of Northern Theater Command Shenyang China; ^4^ Biomedical Research Center, Health Sciences Technology Park University of Granada Granada Spain; ^5^ Ibs. Granada, CIBERfes Granada Spain; ^6^ UGC of Clinical Laboratories Universitu San Cecilio's Hospital Granada Spain; ^7^ Department of Cardiology Zhongshan Hospital, Fudan University Shanghai China; ^8^ Shanghai Institute of Cardiovascular Diseases Shanghai China; ^9^ Department of Laboratory Medicine and Pathology University of Washington Seattle Washington USA

**Keywords:** melatonin, myocardial dysfunction, network pharmacology, PIK3CG, sepsis

## Abstract

Sepsis causes multiple organ injuries, among which the heart is one most severely damaged organ. Melatonin (MEL) alleviates septic myocardial injury, although a systematic and comprehensive approach is still lacking to understand the precise protective machinery of MEL. This study aimed to examine the underlying mechanisms of MEL on improvement of septic myocardial injury at a systematic level. This study integrated three analytic modalities including database investigations, RNA‐seq analysis, and weighted gene co‐expression network analysis (WCGNA), in order to acquire a set of genes associated with the pathogenesis of sepsis. The Drugbank database was employed to predict genes that may serve as pharmacological targets for MEL‐elicited benefits, if any. A pharmacological protein–protein interaction network was subsequently constructed, and 66 hub genes were captured which were enriched in a variety of immune response pathways. Notably, PIK3CG, one of the hub genes, displayed high topological characteristic values, strongly suggesting its promise as a novel target for MEL‐evoked treatment of septic myocardial injury. Importantly, molecular docking simulation experiments as well as in vitro and in vivo studies supported an essential role for PIK3CG in MEL‐elicited effect on septic myocardial injury. This study systematically clarified the mechanisms of MEL intervention in septic myocardial injury involved multiple targets and multiple pathways. Moreover, PIK3CG‐governed signaling cascade plays an important role in the etiology of sepsis and septic myocardial injury. Findings from our study provide valuable information on novel intervention targets for the management of septic myocardial injury. More importantly, this study has indicated the utility of combining a series of techniques for disease target discovery and exploration of possible drug targets, which should shed some light on elucidation of experimental and clinical drug action mechanisms systematically.

## 1 INTRODUCTION

Sepsis is a systemic proinflammatory response evoked by bacterial infection and is commonly associated with the development of multiple organ dysfunction syndrome (MODS). Sepsis is believed the main culprit for high morbidity and mortality in the intensive care unit.^1^ In spite of recent advances in intensive care and the discovery of antibiotics, the mortality rate remains high for severe sepsis, with an ever‐rising incidence rate each year.^2^ Among various organs afflicted by sepsis, the heart is deemed a primary target for sepsis‐induced MODS. Accumulating evidence has denoted the culprit role for myocardial dysfunction in the deterioration of sepsis complications.^3^ The mortality of patients with concurrent sepsis and myocardial dysfunction is approximately 70%–90%, much higher than those patients without myocardial injury (20%).^4^ Therefore, it is pertinent to identify novel and effective therapeutic strategies for septic myocardial injury to improve the prognosis of sepsis.

Melatonin (MEL) is a neuroendocrine hormone synthesized by the pineal gland, with an inherent role in circadian rhythm control.^5^ In addition, MEL is produced in most organs and tissues at much higher levels than the pineal gland—the so‐called extrapineal MEL.^6^ The high levels of extrapineal MEL are closely associated with the ability to eliminate reactive oxygen species (ROS), evoke antioxidant enzyme expression, reduce peroxidase activity, and maintain mitochondrial homeostasis, thereby exerting a powerful anti‐inflammatory and antioxidant effect.^7^, ^8^ Multiple studies have denoted an active role for MEL in various cardiovascular diseases such as hypertension, myocardial ischemia–reperfusion injury, and atherosclerosis.^9^ Notably, MEL also interacts with various pleiotropic pathways, including NOD‐like receptor family pyrin domain containing 3 (NLRP3) signaling,^10^, ^11^ and the mammalian STE20‐like kinase‐1 (Mst1)/c‐Jun N‐terminal kinase (JNK) pathway,^12^ and was suggested to protect against septic myocardial injury.

**FIGURE 1:**
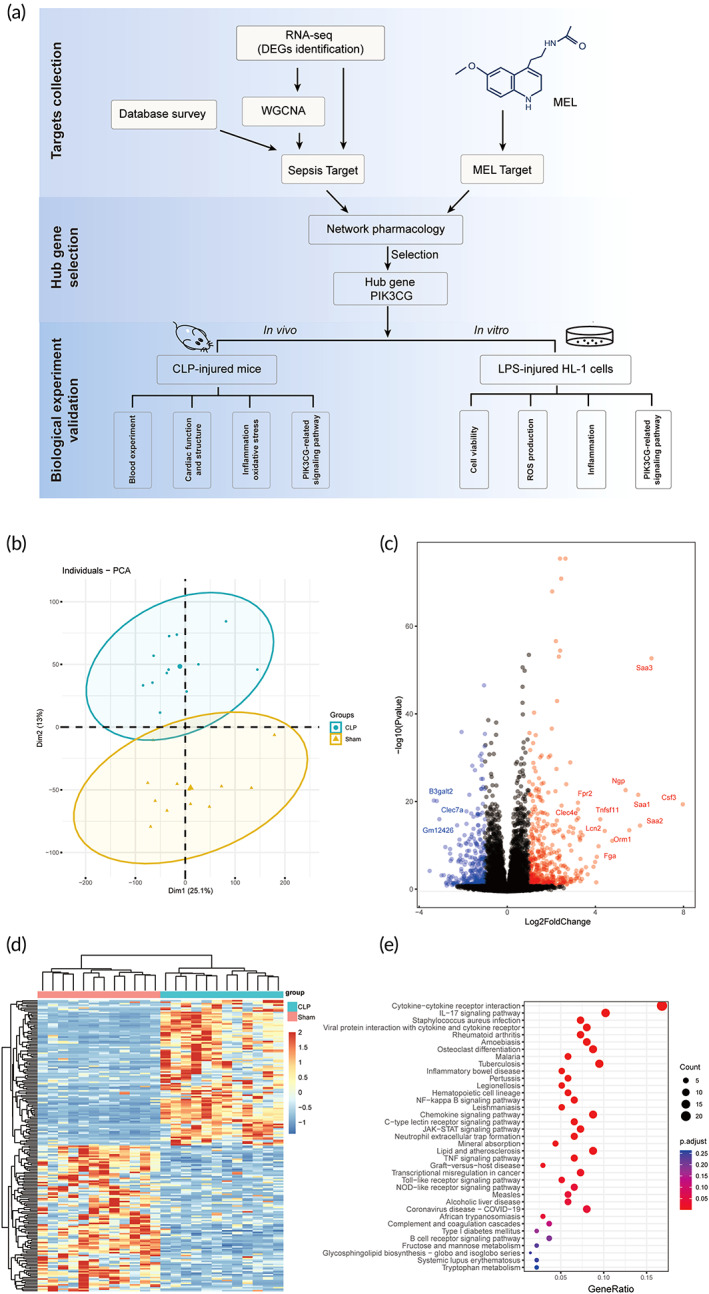
Workflow and gene expression changes between the CLP and Sham group. (a) The workflow includes three parts: target generation, network construction and hub gene selection, and biological experimental verification. (b) Principal component diagrams visualizing protein expression profiles. The samples (*n* = 12 in each group) are plotted along the first two principal component axes (PC1 and PC2). (c) Volcano plot shows the significantly upregulated (red) and downregulated (blue) genes in the CLP group compared to Sham (|Log2 FC| > 1; FDR < 0.01). (d) Heatmap of the top 200 DEGs between the CLP and sham group. The dendrogram describes the hierarchical clustering based on the top 200 DEGs. The top bar indicates the group information: red, sham; green, CLP. The value of the scaled expression is color coded, as shown in the legend on the right. (e) Enriched KEGG pathways for the top 200 DEGs (Fisher's exact test *p* value < 0.05 and FDR‐corrected *p* value < 0.1). CLP, cecal ligation and puncture; DEGs, differentially expressed genes

Although several studies have shown a protective role for MEL in sepsis and septic myocardial injury, the current understanding with regards to pharmacological actions behind MEL is mainly derived from its intervention with common molecules or signaling pathways. A more thorough, systematic and comprehensive approach is urgently needed to unveil the full spectrum behind MEL‐elicited protective mechanisms. Network pharmacology provides a practical strategy for elucidation of potential mechanisms of multicomponent and multitarget drugs by analyzing multiple networks of complex interactions, commonly performed to discover the key targets for disease treatment.^13^, ^14^ Moreover, the weighted gene co‐expression network analysis (WGCNA) and RNA‐seq are also widely used to identify core targets of human diseases.^15^, ^16^ Hence, three strategies (RNA‐seq analysis, WGCNA,^17^ and database search) were conducted in this study to obtain genes associated with the pathogenesis of sepsis (Figure 1a). Then, pharmacological targets for MEL were predicted according to the Drugbank database. Subsequently, a pharmacological network^13^, ^14^ with genes associated with sepsis and putative targets for MEL was constructed to dissect possible therapeutic hub genes. Furthermore, molecular docking simulation and biological experiments (in vitro and in vivo) were employed to verify the possible effect of the identified hub genes in the treatment of sepsis by MEL, if any (Figure 1a). These findings should provide some valuable insights into the molecular mechanisms underscoring sepsis and septic cardiomyopathy. More importantly, we are attempting to optimize a series of methods for the discovery of a disease target and drug action target, which should offer new avenues for exploration of experimental and clinical drug action in a systematic manner.

## 2 RESULTS

### 2.1 RNA‐seq profile and identification of differentially expressed genes

To obtain genes associated with the pathogenesis of sepsis, we performed transcriptome analysis on cecal ligation and puncture (CLP) and sham groups. A total of 24 RNA‐seq libraries were constructed and analyzed, among which 12 biological replicates were included in each group. After removing low‐quality reads, the remaining reads were aligned to mouse genome reference (details in Section 5). A total of 19,080 genes were subjected to batch effect correction (Figure S1) and raw expression data production. Principal component analysis (PCA) results based on these genes showed that the 12 biological replicates from each group were closely clustered together, indicating an acceptable within group variation in replicates (Figure 1b). Moreover, the PCA score plot displayed an observable distinction between CLP and Sham groups, revealing that sepsis caused significant changes in gene expression (Figure 1b). As illustrated in Figure 1c, a total of 778 differentially expressed genes (DEGs) were detected using Deseq2, of which *Saa3*,^18^ *Csf3*,^19^ and *Fpr2* ^20^ have been observed in association with sepsis in previous studies. In addition, a heatmap constructed from the top 200 DEGs (Figure 1d; described in Section 5) also revealed distinct patterns, consistent with the results of the PCA plot (Figure 1b). As expected, kyoto encyclopedia of genes and genomes (KEGG) pathway enrichment analysis demonstrated that the top 200 DEGs were enriched in emergency response related pathways including immune response, signal transduction, and IL‐17 signaling pathway (Figure 1e). Altogether, these results reflected the main changes in transcriptional levels caused by septic myocardial injury. The top 200 DEGs were employed as sepsis pathology‐associated genes for subsequent network pharmacology analysis.

### 2.2 Identification of core genes related to SV, CO, sepsis score, and group in the weighted co‐expression network

**FIGURE 2:**
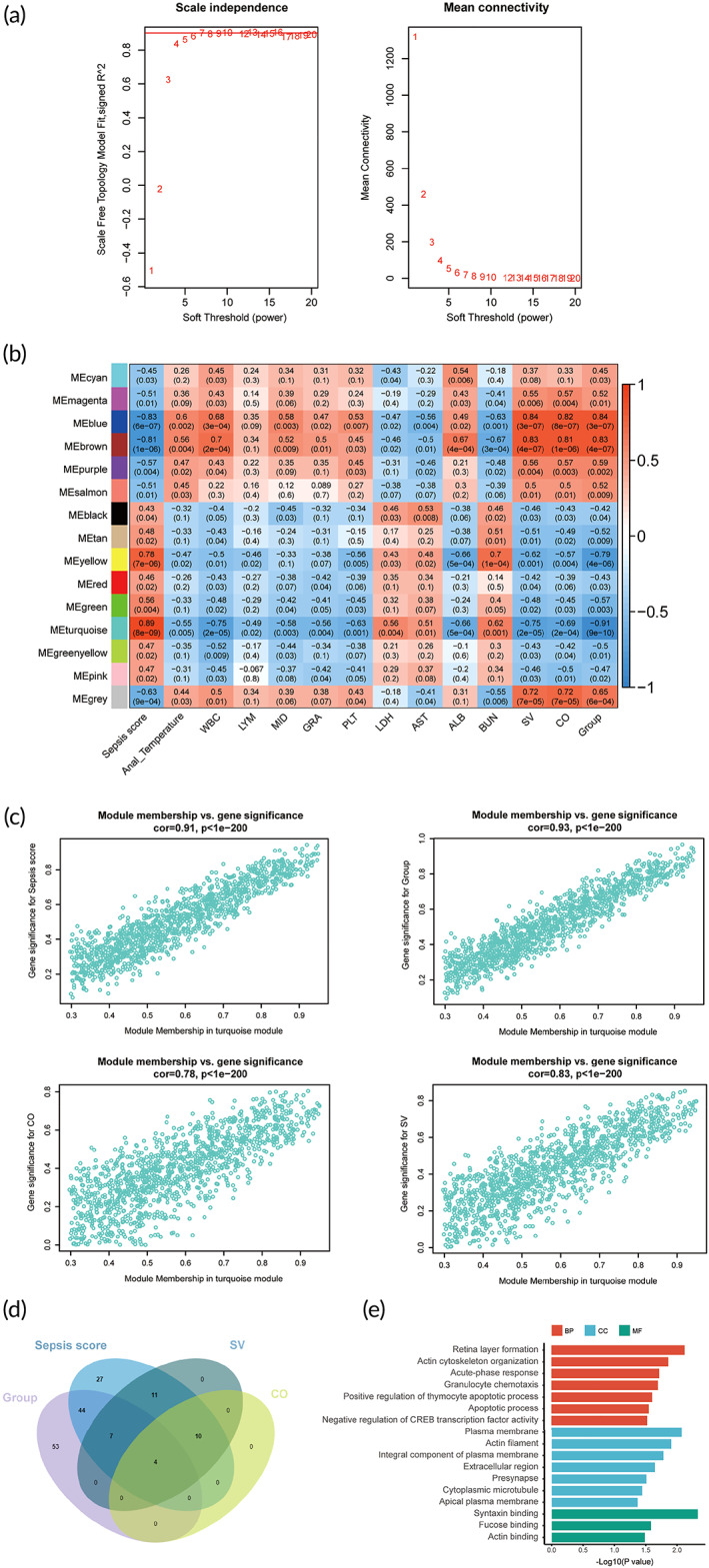
The core genes of WGCNA modules associated with sepsis. (a) The left figure shows the network topology analysis of different soft‐thresholding power *β*. The weighted coefficient *β* is finally set to 8 (*R* ^2^ = 0.85) to construct a signed scale‐free network. The figure on the right illustrates the mean network connectivity under different weighted coefficient. (b) Heatmap of the correlation coefficients between modules and biometric, plasma and echocardiographic parameters of septic mice. Each column refers to a parameter, and each row refers to a ME (the first principal component of each gene module). According to the legend on the right, the matrix is color‐coded by correlation (red, positive correlation; blue, negative correlation). The Pearson correlation coefficient is shown in the rectangle and the *P value* is shown in parentheses. Module turquoise is significantly correlated with parameters SV, CO, group and sepsis score. (c) According to the threshold (|MM| > 0.8, |GS| > 0.8; details in Section 5), the core genes correlated with SV, CO, group, and sepsis score are extracted from the module turquoise. The veen diagram of these core genes is drawn in (d), and the GO terms enrichment result is shown in (e). AST, aspartate aminotransferase; BUN, blood urea nitrogen; CO, cardiac output; CK, creatine kinase; GRA, granulocytes; GS, Gene significance; LDH, lactic dehydrogenase; LYM, lymphocytes; MEs, module eigengenes; MID, middle cells; MM, module membership; PLT, platelets; SV, stroke volume; RBC, red blood cells; WBC, white blood cells; WGCNA, weighted gene co‐expression network analysis

WGCNA is generally used to identify modules and candidate marker genes related to external sample traits and has been successfully performed in various biological entities, such as cancer and mouse genome.^21^, ^22^ Here in this study, WGCNA was applied to quest genes related with biometric, plasma and echocardiographic parameters of septic mice (see Section 5 for details), in an effort to identify core candidate targets by structuring a scale‐free gene co‐expression network. The normalized count matrix of a total of 5699 genes after quality control was used as input data. The power of *β* = 8 (scale free *R* ^2^ = 0.85) was chosen as the soft‐thresholding parameter to ensure a scale‐free network (Figure 2a). Fifteen modules were eventually identified via the average linkage hierarchical clustering (Figure S2a,b). The turquoise module displayed a significant negative correlation with group (−0.91, *p* = 9e‐10), stroke volume (SV) (−0.75, *p* = 2e‐05), and cardiac output (CO) (−0.69, *p* = 2e‐04), and a positive correlation with sepsis score (0.89, *p* = 8e‐09) (Figure 2b). Subsequently, we extracted genes in the turquoise module that were positively correlated with sepsis score although negatively correlated with SV, CO, and group, based on the threshold values of module eigengenes (MEs) and gene significance (GS) (Figure 2c) (details in Section 5). A total of 156 candidate genes were selected, with a high proportion shared between the four gene sets (Figure 2d) and showed significantly enriched in pathways related to the acute phase of immune response (Figure 2e). The core genes were selected as part of sepsis related genes to do further network pharmacology analysis.

### 2.3 Pharmacological network analysis and hub targets screening of MEL for protection against infectious myocardial injury

**FIGURE 3:**
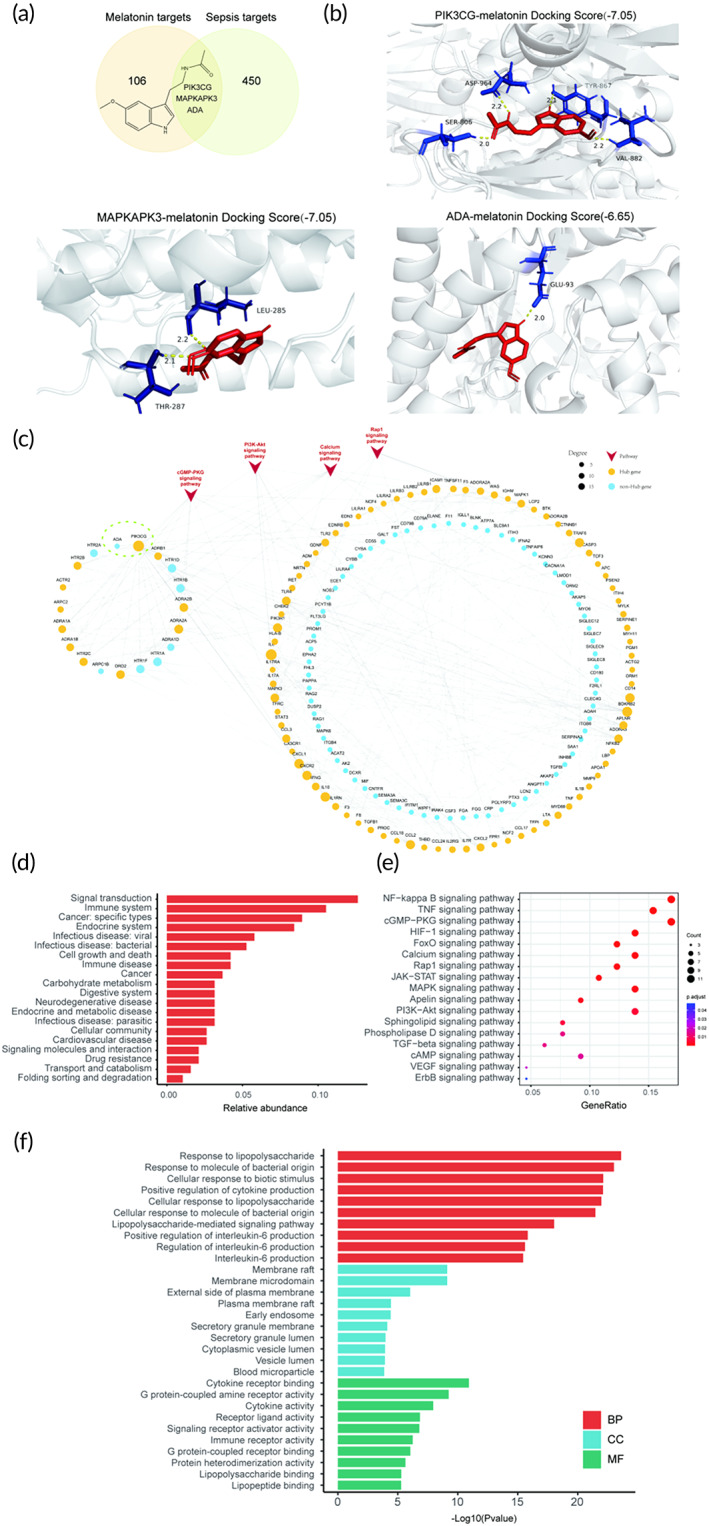
The molecular basis of the protective effect of MEL on sepsis. (a) Venn diagram depicting the overlap between sepsis‐related targets and MEL putative targets. The background image shows the chemical structure of MEL. (b) The molecular docking simulation of the binding pattern of MEL with ADA, PIK3CG, and MAPKAPK3. MEL, melatonin; IL‐6, Interleukin 6. (c) The interaction network describes the connections between MEL putative targets (nodes in the left circle) and sepsis‐related genes (nodes in the right concentric circle). The nodes circled by dotted lines are genes shared by sepsis‐related genes and MEL putative targets (MAPKAPK3 has no link). Yellow and blue nodes symbolize hub genes and non‐hub genes, respectively. Node size is ranked according to its network centrality, and PIK3CG is the second largest node in the network, behind the IL‐6. The red triangle nodes refer to the top five KEGG pathways (sorted by rich factor) of the signal transduction classification pathways enriched by the hub genes. The bar graph (d) shows the classification of KEGG pathways enriched by these hub genes, the dot plot (e) shows the pathways of signal transduction classification, and the bar chart (f) exhibits the enriched GO terms

A total of 109 known sepsis‐related genes were collected from multiple databases (details in Section 5). After the integration and deduplication of the top 200 DEGs, 156 core genes and 109 known genes, in total, 453 sepsis‐related genes were obtained (Figure 3a and Table S2). This study predicted a total of 109 drug targets (Figure 3a and Table S3) based on the Drugbank^23^ database using the structure of MEL, which yielded three shared sepsis‐related genes (PIK3CG, ADA, and MAPKAPK3, as shown in Figure 3a). Molecular docking showed the strongest binding for PIK3CG with MEL compared to the other two targets (Figure 3b). To elaborate the relationship of the MEL's putative targets with sepsis‐related genes, the interaction network was built based on the STRING^24^ database and visualized by the Cytoscape software (Figure 3c). The 66 hub genes were screened by classifying the hub importance according to the values of the network topological centralities (degree, betweeness, and closeness centralites) (Table S5). These aforementioned 66 hub genes were enriched into three functional groups (BP, CC, and MF) of GO terms (Figure 3f and Table S6; only displayed the top 10 terms in each group). Hub genes in the BP group were mainly enriched in response to lipopolysaccharide, response to molecule of bacterial origin, and positive regulation of cytokine production (Figure 3f, Table S6). The genes in the CC group were mainly focused on membrane raft and secretory granule lumen, while the genes in the MF group were heavily resided in cytokine activity, immune receptor activity, receptor ligand activity, and signaling receptor activator activity (Figure 3f, Table S6). Moreover, the enriched KEGG pathways were classified, which demonstrated an essential involvement in signal transduction and immune response (Figure 3d and Table S7), including PI3K‐AKT, Rap1 signaling, NF‐kappa B signaling, and FoxO signaling pathways (Figure 3e, Table S8). Functional analysis of these hub genes suggested that MEL ameliorated myocardial injury in sepsis by multiple targets and pathways.

Among these hub genes, PIK3CG with high centrality values, participated in PI3K‐AKT signaling pathway, and cGMP‐PKG signaling pathway (Figure 3c, Tables S5 and S8). In addition, PIK3CG showed the strongest binding affinity with MEL among the three common targets shared between MEL targets and sepsis targets (Figure 3b). Hence, we propose that MEL plays a protective role in sepsis by regulating immune‐related pathways, mainly through the PIK3CG‐based PI3K‐AKT pathway.

### 2.4 The effects of MEL on survival rate, sepsis score, and anal temperature in CLP‐injured mice

**FIGURE 4:**
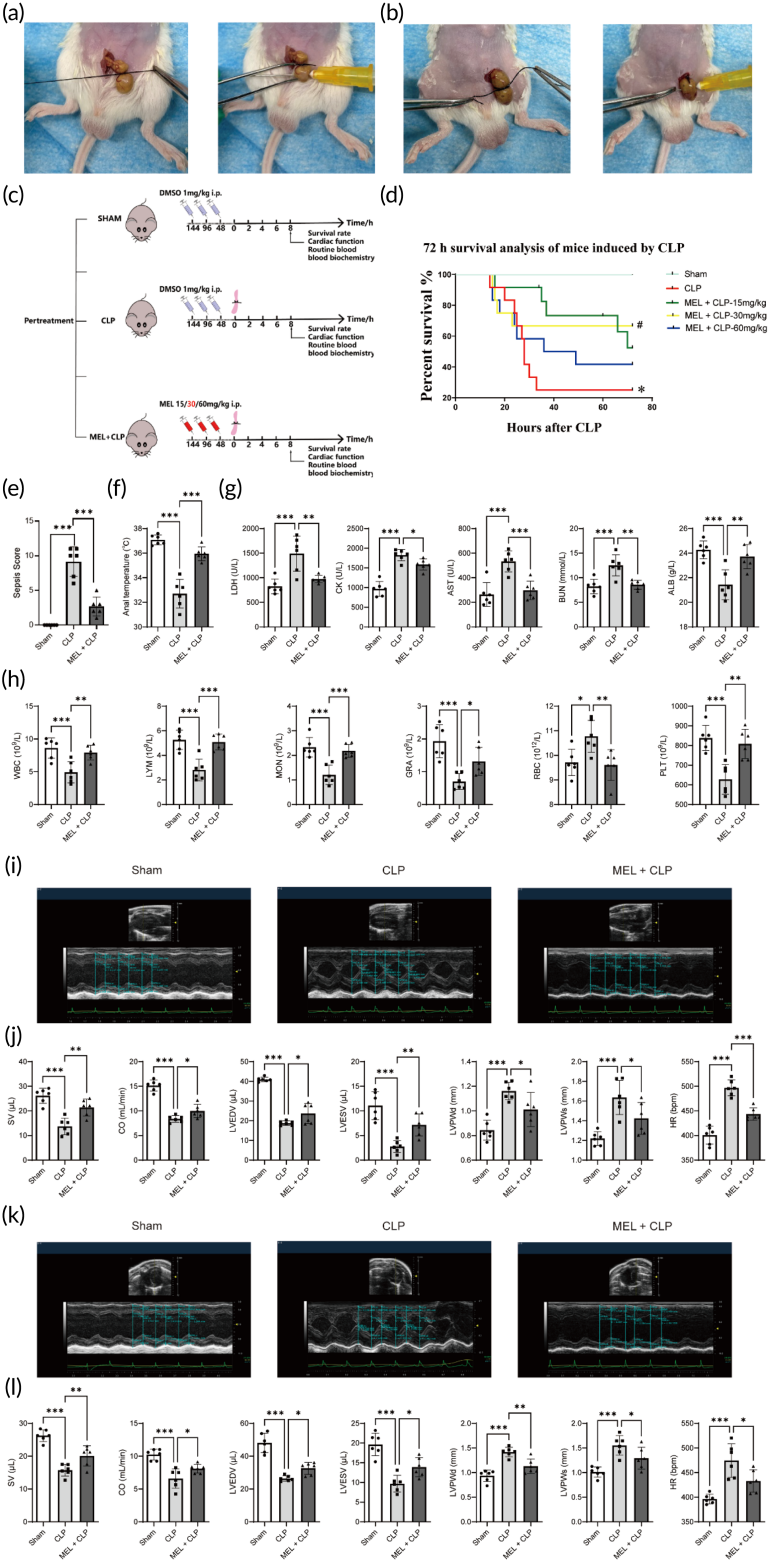
The effects of MEL on survival rate, sepsis score, anal temperature, blood routine parameters, blood biochemical parameters, and cardiac function in CLP‐injured mice. (a) CLP model protocol. (b) Aggravated CLP model protocol. (c) A schematic diagram of the experimental group and protocol. (d) The 72 h survival analysis of mice induced by CLP, *n* = 10. (e) Sepsis score, *n* = 6. (f) Anal temperature, *n* = 6. (g) Blood biochemical parameters. (h) Blood routine parameters. (i) Representative echocardiography images of the long axis. (j) Statistical graph of SV, CO, LVEDV, LVESV, LVPWd, LVPWs, and HR. (k) Representative echocardiography images of the short axis. (l) Statistical graph of SV, CO, LVEDV, LVESV, LVPWd, LVPWs, and HR. *p < 0.05, **p < 0.01, and ***p < 0.001 compared with the CLP group (d–l); ns, nonsignificant. Using one‐way ANOVA and Tukey's multiple comparisons. CLP, cecal ligation and puncture; CO, cardiac output; HR, heart rate; LVPWd, left ventricular posterior wall thickness of diastole period; LVEDV, left ventricular diastolic volume, LVESV, left ventricular systolic volume; LVPWs, left ventricular posterior wall thickness of systole period; MEL, Melatonin; SV, stroke volume

To validate the hub genes detected, both in vivo and in vitro experiments were performed. CLP is one of the most widely used model for experimental sepsis. The establishment of CLP model mainly consists of two steps: cecal ligation and puncture (Figure 4a,b). Mice were given different levels of MEL (15, 30 and 60 mg/kg) + DMSO or 1 ml/kg DMSO to observe survival rate in the CLP group (for 72 h). The results showed that CLP led to a high mortality rate (nearly 75%). However, MEL overtly improved the survival rate. Among various MEL groups, 30 mg/kg MEL pretreatment exhibited an obvious protective effect (vs. CLP group, Figure 4d; *p* < 0.05). Therefore, this level of MEL was used for the remaining studies. Subsequently, sepsis score and anal temperature were monitored 8 h post‐CLP. In this study, the MSS system was introduced to monitor experimental mice based on their physical appearance, level of consciousness, activity, response to stimuli, eyes, respiration rate and quality (from 0 to 4 points for each criteria).^25^ The results showed that CLP significantly increased sepsis score, the effects of which were reversed by MEL treatment (Figure 4e). As shown in Figure 4f, MEL significantly increased the anal temperature in CLP‐injured mice (vs. CLP group, *p* < 0.05).

### 2.5 The effects of MEL on blood routine parameters, blood biochemical parameters, cardiac function, and myocardial structure in CLP‐injured mice

**FIGURE 5:**
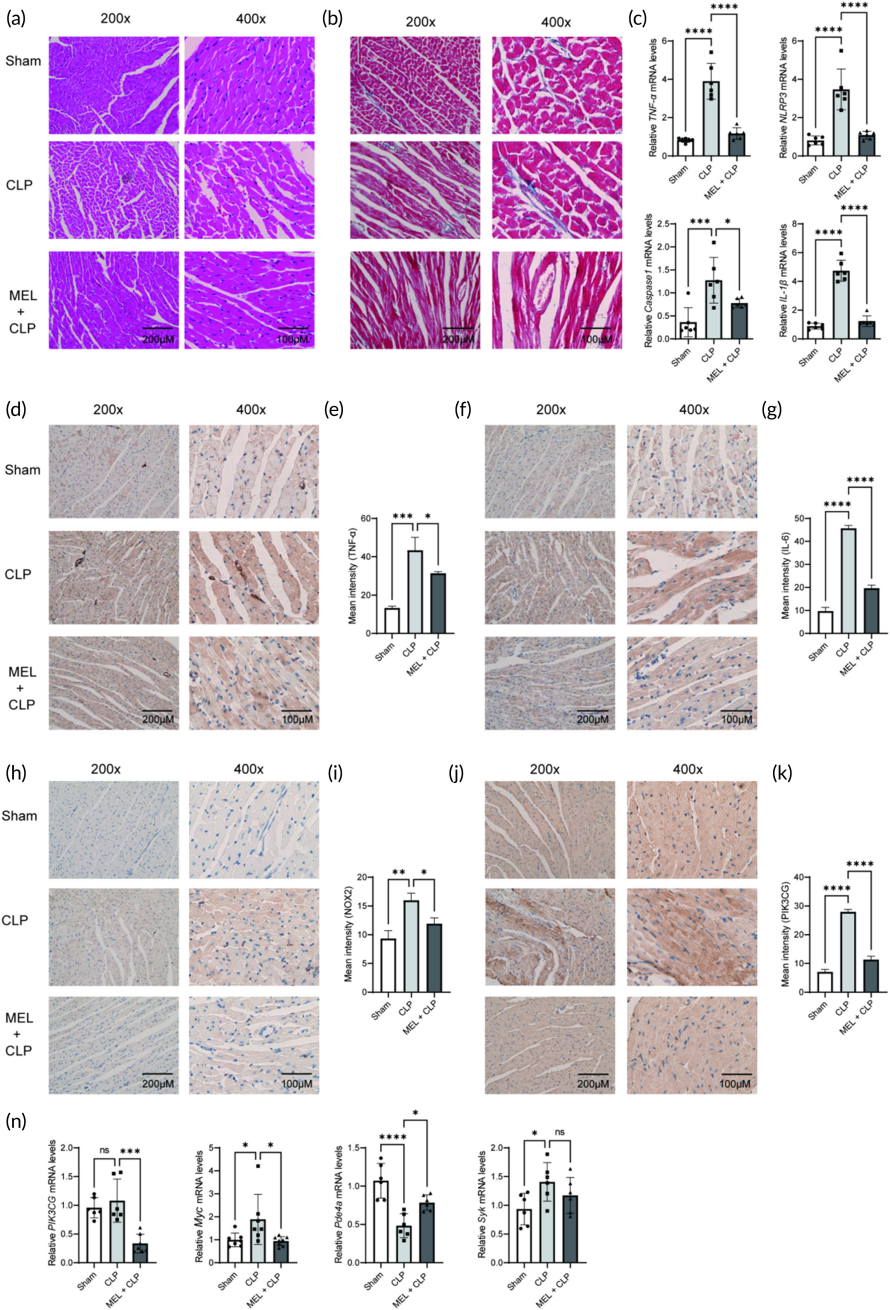
The effects of MEL on myocardial structure, inflammatory response, oxidative stress, and PIK3CG‐related signaling pathway in CLP‐injured mice. (a) H&E staining of myocardium. (b) Masson staining of myocardium. (c) qRT‐PCR analysis of NLRP3, TNF‐α, IL‐1β, and Caspase‐1 mRNA by normalizing to β‐actin. (d) Representative photographs of TNF‐α IHC staining. (e) Statistical graph of TNF‐α IHC staining. (f) Representative photographs of IL‐6 IHC staining. (g) Statistical graph of IL‐6 IHC staining. (h) Representative photographs of NOX2 IHC staining. (i) Statistical graph of NOX2 IHC staining. (j) Representative photographs of PIK3CG IHC staining. (k) Statistical graph of PIK3CG IHC staining. (l) qRT‐PCR analysis of *PIK3CG*, *Pde4a*, *Syk*, and *Myc* mRNA by normalizing to β‐actin. *p < 0.05, **p < 0.01, and ***p < 0.001 compared with the CLP group; ns, nonsignificant; *n* = 6. Using one‐way ANOVA and Tukey's multiple comparisons. CLP, cecal ligation and puncture; H&E, hematoxylin–eosin; IHC, immunohistochemistry; IL‐6, interleukin 6; MEL, melatonin; NLRP3, NOD‐like receptor family pyrin domain containing 3; TNF‐a, tumor necrosis factor‐a

Then, blood routine and blood biochemical parameters were evaluated in CLP‐injured mice. Compared with CLP group, MEL remarkably increased levels of white blood cells (WBC), lymphocytes (LYM), monocytes (MON), granulocytes (GRA), and platelets (PLT), decreased the level of red blood cells (RBC) (Figure 4h; *p* < 0.05). In addition, levels of blood biochemical parameters (lactic dehydrogenase (LDH), creatine kinase (CK), blood urea nitrogen (BUN), and aspartate aminotransferase (AST)) were significantly increased, and level of albumin (ALB) was decreased after CLP injury, the effects of which were reverted by MEL treatment (Figure 4g; *p* < 0.05). Myocardial function was assessed using echocardiography. As shown in Figure 4i–l, CLP resulted in severe myocardial injury, as evidenced by decreased SV, CO, left ventricular diastolic volume (LVEDV), left ventricular systolic volume (LVESV) as well as increased left ventricular posterior wall thickness of systole period (LVPWs), left ventricular posterior wall thickness of diastole period (LVPWd), and heart rate (HR) (vs. Sham group, *p* < 0.05), the effects of which were mitigated by MEL (vs. CLP group, *p* < 0.05). Besides, H&E staining showed obvious structural abnormalities and myocardial fibrosis in CLP‐injured myocardium, while MEL treatment remarkably restored these CLP‐induced pathological changes (Figure 5a,b). These results suggest that MEL possesses a protective effect against septic myocardial injury.

### 2.6 The effects of MEL on inflammatory response, oxidative stress, and PIK3CG‐related signaling pathway in CLP‐injured mice

NLRP3 is a crucial mediator of initiating immune response and inflammasome formation, and once activated, it directly promotes the production of IL‐1β, IL‐18, and Caspase‐1. As shown in Figure 5c, the qRT‐PCR assay demonstrated that CLP injury significantly increased *NLRP3*, *IL‐1β*, *TNF‐α*, and *Caspase‐1* mRNA expressions (vs. Sham group, *p* < 0.05). However, MEL treatment reversed these culprit effects (vs. CLP group, *p* < 0.05; Figure 5c). Moreover, the IHC staining of inflammatory markers (IL‐6 and TNF‐α) and oxidative stress markers (NOX2) were also performed in myocardium. As expected, MEL significantly attenuated the inflammatory response and oxidative stress, as indicated by expression of IL‐6, TNF‐α, and NOX2 (vs. CLP group, *p* < 0.05; Figure 5d–i). Subsequently, this study attempted to investigate the underlying mechanisms through which MEL protected against septic myocardial injury. This study identified a key PIK3CG‐related signaling pathway. The results confirmed that CLP injury increased expression of *PIK3CG*, *Myc*, and *Pde4a* mRNA, the effect of which was reversed by MEL (Figure 5l; *p* < 0.05). Moreover, the IHC staining also confirmed that MEL decreased PIK3CG expression (vs. CLP group, *p* < 0.05; Figure 5j,k). These results suggested that PIK3CG‐based PI3K‐AKT pathway may be involved in the protective effect of MEL in septic myocardial injury.

### 2.7 The effects of MEL on cell viability, ROS production, and PIK3CG‐related signaling pathway in LPS‐injured cardiomyocytes

**FIGURE 6:**
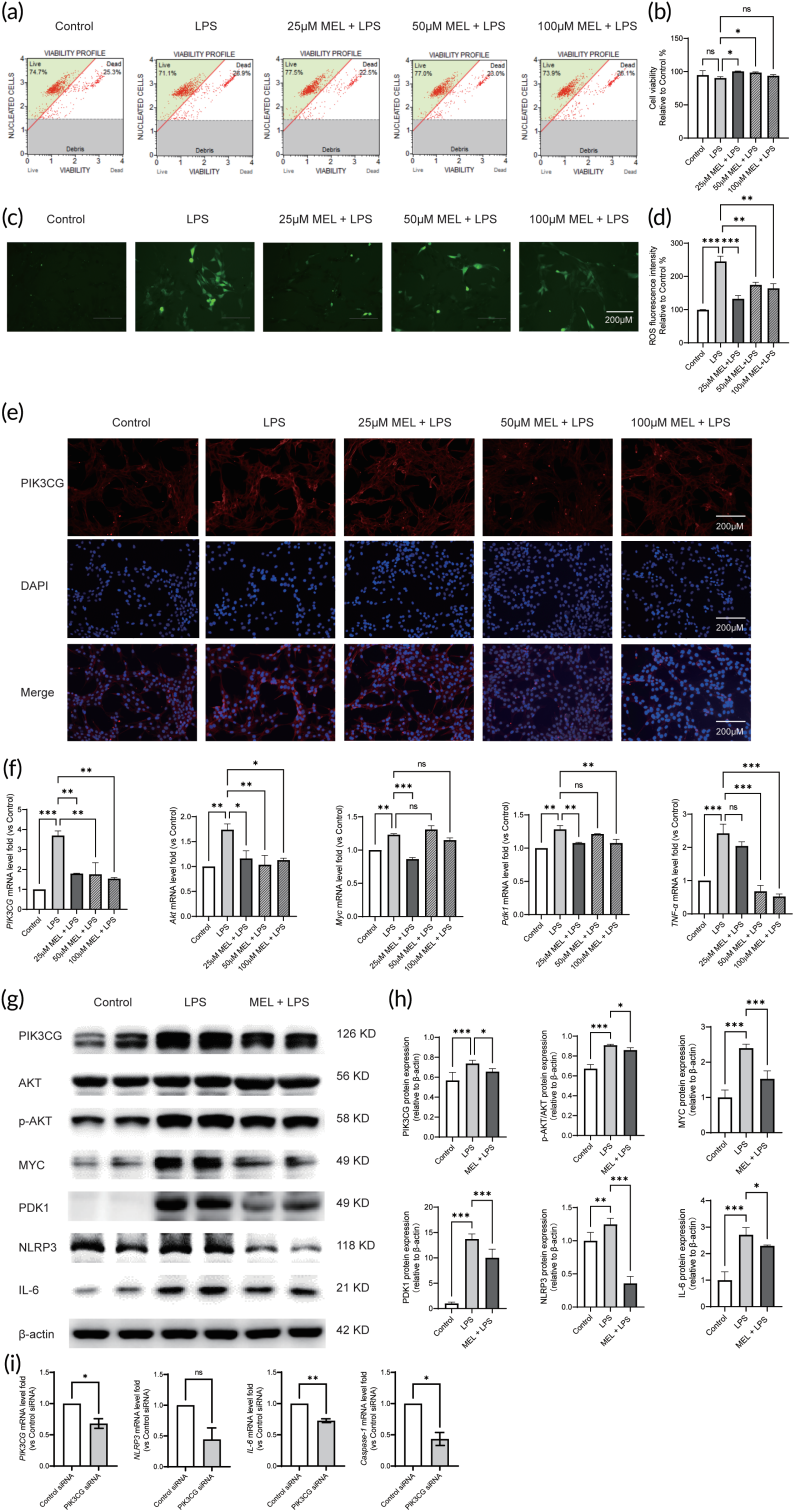
The effects of MEL on cell viability, ROS production, and PIK3CG‐related signaling pathway in LPS‐injured cardiomyocytes. (a) HL‐1 cells were treated with different concentrations of MEL (3 h) before exposure to LPS (3 h), followed by detection of cell viability. (b) Statistical graph of cell viability. (c) Intracellular ROS of LPS‐injured HL‐1 cells. (d) Statistical graph of intracellular ROS. (e) Representative photographs of PIK3CG DAPI staining. Using a specific anti‐PIK3CG antibody (red), DAPI to recognize the nucleus (blue), and the merged image. (f) qRT‐PCR analysis of *PIK3CG*, *Akt*, *Myc*, *Pdk1*, and *TNF‐α* mRNA by normalizing to β‐actin. (g) The representative images of PIK3CG, PDK1, p‐AKT/AKT, MYC, NLRP3, IL‐6 detected by Western blot. (h) Quantitative analysis of Western blot by normalizing to β‐actin. (i) qRT‐PCR analysis of PIK3CG, NLRP3, Caspase‐1, and IL‐6 mRNA by normalizing to β‐actin. *p < 0.05, **p < 0.01, and ***p < 0.001 compared with the LPS group; ns, nonsignificant; *n* = 6. Using one‐way ANOVA and Tukey's multiple comparisons. LPS, lipopolysaccharide; MEL, melatonin; PDK1, 3‐phosphoinositide‐dependent protein kinase‐1; ROS, reactive oxygen species; TNF‐a, tumor necrosis factor‐a

HL‐1 cells were pretreated with various concentrations of MEL (25, 50, and 100 μM) prior to exposure to LPS for 3 h. The results showed that MEL exerted the most significant protective effect against LPS‐injured cells, as evidenced by increased cell viability and a decreased intracellular ROS production (vs. the LPS group, Figure 6a–d; *p* < 0.05). Moreover, the qRT‐PCR assay demonstrated that LPS treatment significantly increased *Akt*, *IL‐6*, *TNF‐α*, *Myc*, and *Pdk1* mRNA expressions in LPS‐injured HL‐1 cells, the effect of which was abrogated by MEL (vs. the LPS group, Figure 6f; *p* < 0.05). Immunofluorescence and western blot results also indicated that MEL countered LPS‐induced hyperactivated PIK3CG signaling cascade (vs. the LPS group, Figure 6e,g,h; *p* < 0.05). Moreover, the levels of p‐AKT/AKT, MYC, PDK1, NLRP3, and IL‐6 rose significantly after LPS injury, the effects of which were reversed by MEL (Figure 6g,h; *p* < 0.05). Subsequently, PIK3CG was silenced by small interfering RNAs (siRNAs). The qRT‐PCR assay demonstrated that PIK3CG siRNA significantly decreased PIK3CG, NLRP3, Caspase‐1, and IL‐6 mRNA expressions but increased Bax mRNA expressions in HL‐1 cells (vs. the Control siRNA group, Figure 6i; *p* < 0.05).

## 3 DISCUSSION

**FIGURE 7:**
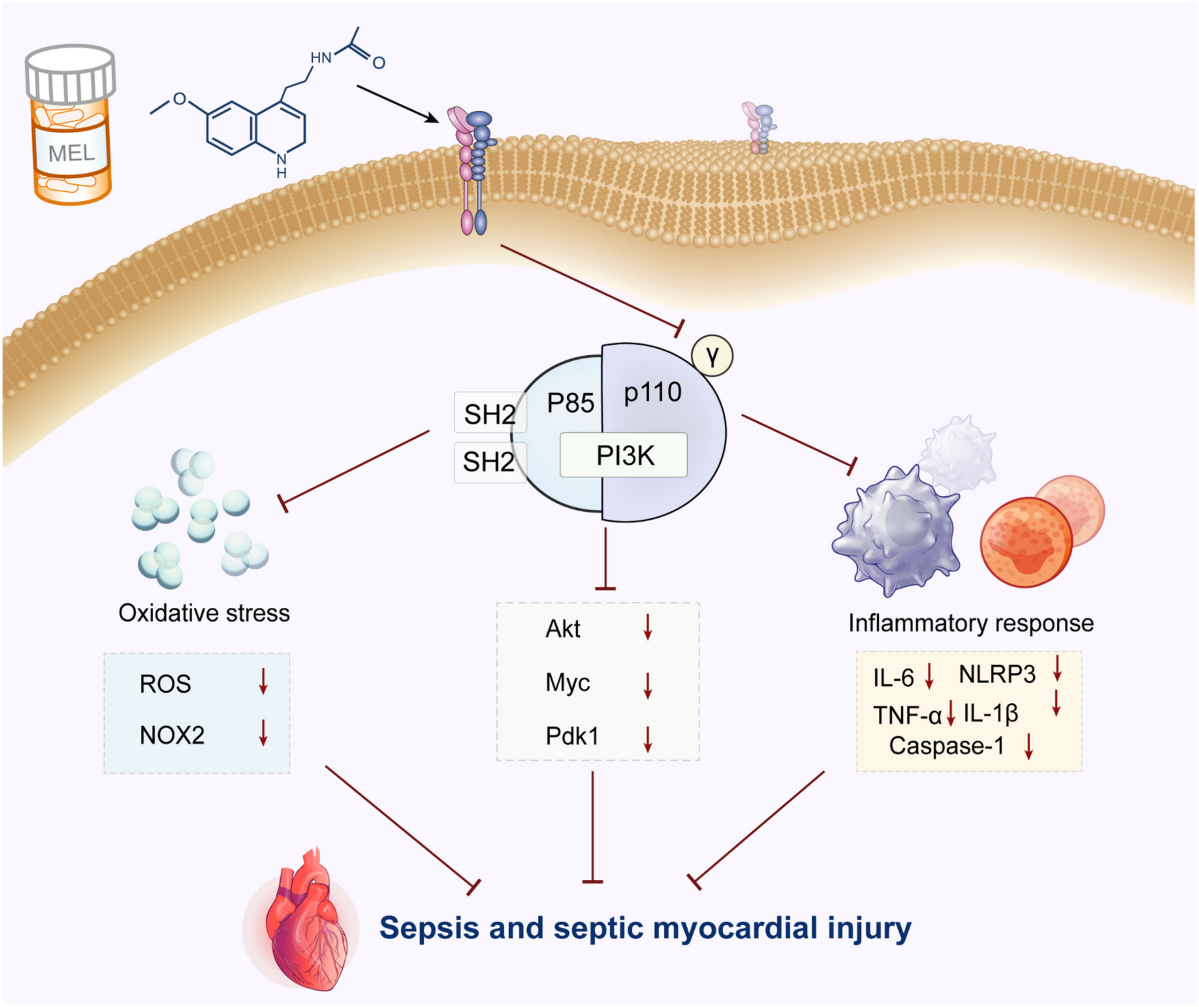
The mechanisms of MEL protecting against septic myocardial injury via inhibiting PIK3CG‐related signaling pathway. MEL, melatonin

In this study, we employed a combination of RNA‐seq analysis, WGCNA, pharmacological network construction, and molecular docking to identify genes and pathways involved in the treatment of septic myocardial injury. First, the 453 sepsis‐related genes were obtained by merging the top 200 DEGs from RNA‐seq analysis, 109 known targets from database, and 156 core genes from WGCNA. Second, 109 targets of MEL were predicted using the Drugbank database. Based on a pharmacological network constructed by drug targets and disease genes, PIK3CG was identified as one essential candidate target as supported by molecular docking. As expected, in vivo and in vitro studies also confirmed that MEL exerts a pronounced cardioprotective effect against septic myocardial injury through inhibition of PIK3CG‐related signaling pathway (Figure 7).

PI3K is an important component of the PI3K‐Akt intracellular signaling pathway, consisting of regulatory subunit (p85) and catalytic subunit (p110). PI3K is reported to be involved in cell proliferation, apoptosis, migration, and inflammation among other pathophysiological processes, affecting the onset and development of a variety of cardiovascular diseases.^26^ There are four types of catalytic subunits of PI3K, namely p110α, β, δ, and γ. Among them, p110α, β, and δ are involved in immunity and inflammation.^27^, ^28^ PI3Kγ/p110γ, also known as PIK3CG, is a key regulator molecule in the pathological process of inflammation and oxidation, and thus plays a critical role in a variety of cardiovascular diseases.^26^ For example, blood pressure can be declined in a dose‐dependent fashion through inhibition of PI3KCG in mice.^29^ Moreover, the deficiency of PIK3CG protects anthracycline‐induced cardiotoxicity and decreases tumor growth.^30^ However, whether PIK3CG participates in septic myocardial injury is essentially unknown. Hence, we performed the network pharmacological analysis and molecular docking simulation and found that PIK3CG was a potential core target of MEL for the treatment of septic myocardial injury. Importantly, in vivo and in vitro experiments showed that MEL alleviated sepsis and septic myocardial injury by inhibiting PIK3CG‐related signaling. Hence, we present evidence here for optimization of a series of methods for discovering disease and drug targets, to identify and verify involvement of PIK3CG in MEL‐offered benefit against sepsis myocardial injury, thus unveiling a key role for PI3K family members in sepsis.

As mentioned above, a systematic and comprehensive approach is urgently needed to explore the mechanisms of MEL in the protection against septic myocardial injury. Network pharmacology provides a practical strategy for elucidating the potential mechanisms of multicomponent and multitarget agents by analyzing various networks of complex interactions, which is widely used to discover key pathways and target molecules for disease treatment.^13^ However, target information obtained by database retrieval is limited, which compromises the accuracy and comprehensiveness of the mechanisms research.^13^ To eliminate this limitation, three strategies were adopted in this study to characterize genes related to sepsis. First, recognized databases (HPO, TTD, TCMIP, DrugBank, DisGeNET, TCMSP) were used to search for sepsis correlated target genes (109 item genes). Second, genes with significant transcriptional change in CLP group (Top 200 DEGs) were utilized to characterize targets related to sepsis. Subsequently, WGCNA was conducted to identify co‐expression modules and module‐associated genes, defining sepsis‐related targets (156). Thereafter, a multisource, comprehensive set of sepsis‐related genes was obtained by integrating the aforementioned three sets of data, offering information fundamental for the analysis of network pharmacology. The “sepsis‐related gene‐MEL‐putative target” interaction network was constructed using the well‐supplied disease genes and drug putative targets. The hub genes scanned in this network have been partially identified (e.g., *Saa3* and *Fpr2*) as potential targets of septic myocardial injury in previous studies.^31^, ^32^ Moreover, the expression of PIK3CG, Myc and Syk hub genes were significantly increased in CLP‐injured mice and LPS‐injured cardiomyocytes, the effects of which were nullified by MEL treatment. The aforementioned evidence reported in our current study primarily shows the reliability of this method. Notably, this represents the first time that differential expression analysis has been combined with WGCNA to provide a wealth of disease data resources, construct a complex pharmacological network, and reveal the possible targets and mechanisms of MEL in the treatment of sepsis.

## 4 CONCLUSION AND LIMITATION

This study clarified the mechanisms of MEL intervention in septic myocardial injury involved multiple targets by integrating multiple approaches. Additionally, PIK3CG‐governed signaling cascade plays an important role in the etiology of sepsis and septic myocardial injury. However, the regulation pattern and upstream/downstream pathways of PIK3CG need to be further investigated. This finding provides valuable information on novel intervention targets for the management of septic myocardial injury.

## 5 METHODS

### 5.1 RNA‐sequencing and data analysis

In this study, a total of 24 male BALB/c mice were divided into sham and CLP groups. Myocardium (100 mg) was collected at post‐8 h, and TRIzol reagent (Invitrogen Life Technology Co., Ltd, USA) was employed to extract total RNA from mouse tissues. Quality and quantity of RNA extracts were checked using a Bioanalyzer 2200 (Agilent Technologies Inc., Shanghai, China). The cDNA libraries were sequenced using a HiSeqXTen (Illumina) platform and finished the sequencing in three batches. Raw reads were mined for clean reads by removing the adaptor sequences, reads with >5% ambiguous bases (noted as *N*), and low‐quality reads containing more than 20% of bases with qualities of <20. Hisat2 software was employed to align clean reads to mouse genome (version: mm10 NCBI). The SVA/Combat software^33^ was utilized to alleviate any potential batch effects due to multiple sequencing runs using a raw count matrix (Figure S1). The EdgeR package in R language was applied to normalize mRNA sequencing data. The DEGs between CLP and Sham groups were obtained using Deseq2 and the threshold was set as follows: (i) Fold Change > 2 or <0.5; (ii) FDR‐corrected *p* < 0.05. The heat map and volcano map were generated based on the top 200 DEGs (top 100 upregulated genes and top 100 downregulated genes) in the CLP group using the ggplot2 package in R language.

### 5.2 GO terms and KEGG enrichment

To illustrate biological implications behind different gene sets including WGCNA core genes, DEGs, and hub genes, GO terms and KEGG pathway enrichment analyses were applied using the ClusterProfiler^34^ package with cut‐offs of *p* value < 0.05 in the Fisher's exact tests and an FDR‐corrected *p* value < 0.1.

### 5.3 Weighted gene co‐expression network analysis

Quality‐controlled count matrix was applied to identify significant gene modules and determining the core genes related to biometric, plasma and echocardiographic parameters of septic mice using the WGCNA package^35^ in R. The filtering criteria for the expression data of the Sham and Sepsis group are as follows: (1) filter out the data whose sum of the expression levels of all samples is less than 10; (2) filter out the data whose coefficient of variation exceeds 50 in the sham group and sepsis group, respectively. The mouse biometric, plasma, and echocardiographic parameters were collected at 8 h post‐CLP (Table S4). A weighted adjacency matrix was constructed based on the power adjacency *a*_*ij*_ = |cor(*x*_*i*_,*x*_*j*_)|^*β*^.^35^ The cor operator denotes the Pearson's correlation coefficient in levels between gene *i* and gene *j*. The power of *β* (weighted coefficient) was set to be 8 (*R* ^2^ = 0.85) to establish a signed scale‐free network. Next, a topological overlap matrix (TOM) was derived using the adjacency matrix data and the dynamic tree cut algorithm was performed to identify gene modules (which corresponded to colored branches of dendrogram). To decipher connections between gene modules and biometric, plasma and echocardiographic parameters of septic mice, Pearson's correlation coefficients between mouse traits and the module eigengene (ME, the first principal component of each gene module) were calculated.^35^

In order to screen the core gene candidates, modules associated with the traits of interest, in our case “SV,” “CO,” “group,” and “sepsis score” were further considered for the intranodal analysis. The significance threshold of module selection was set as *p* = 0.05. Within a given module, gene significance (GS) described the relationship between gene expression profile and each trait. Module membership (MM) was defined as the correlation between the gene expression profile and module eigengene. The core genes were picked out according to the strict cut‐off criteria |MM| > 0.8, |GS| > 0.8. For details in each procedure, please refer to the previous work of Langfelder and Horvath.^35^

### 5.4 Generation of MEL putative targets and sepsis‐related genes

Structural information of MEL was acquired from the PubChem database (https://pubchem.ncbi.nlm.nih.gov/). Based on structural information, MEL putative targets were surveyed based on DrugBank 5.0 with similarity >0.6.^23^ Sepsis‐related genes were partially collected from known database TCMSP^36^ (https://www.tcmsp-e.com/) and TCMIP^37^ (http://www.tcmip.cn/TCMIP/index.php/Home/), which integrates information from the DisGeNET database,^38^ Human Phenotype Ontology (HPO),^39^ DrugBank database,^23^ and Therapeutic Target Database (TTD).^40^ The search updated in November 24, 2021 and the search term was set as “sepsis.” The inconsistent gene IDs of different resources were manually inspected and converted into Official Gene Symbols. In reference to the previous study,^41^ the top 200 DEGs (top 100 upregulated and top 100 downregulated genes), as representatives of highly variable genes, were selected as sepsis‐associated genes. In addition, a portion of 156 core genes picked from sepsis‐related module in the section of WGCNA was also included. Subsequently, a total of 453 genes were collected as sepsis‐related genes following integration and de‐duplication of the three sets (Table S2).

### 5.5 Network construction

The “sepsis‐related gene‐MEL‐putative target” interaction network was analyzed using the public database STRING v11.5^24^ between sepsis‐related genes and putative targets of MEL. Official gene symbols of sepsis‐related genes and MEL‐putative targets were used as input to construct the network. The STRING parameter is 0.4 of confidence level and is 0.05 of FDR stringency. Network was visualized through Cytoscape software^42^ (Version 3.5.1). To evaluate centrality, three topological characteristics^42^ (degree, betweenness, and closeness) were calculated for each node in the network. For a given node, degree referred to the number of links to it, betweenness was the number of all shortest paths through it, and closeness corresponded to the inverse of the average shortest path length from it to all other nodes in the network. A node with larger values of the topological characteristics indicates a more important role in the interaction network. A hub gene was defined as a node whose degree, betweenness, and closeness values exceeded the median values of the overall nodes. KEGG pathway enriched by the hub genes was also displayed in the network.

### 5.6 Molecular docking

The structures of proteins (PIK3CG, ADA, and MAPKAPK3) were obtained from the RSCB PDB database (RCSB PDB database https://www.rcsb.org/). The structural information of MEL was obtained from the PubChem database, and then the sdf format was converted to the mol2 format using the OpenBabel v3.1.1 software. The proteins were preprocessed with AutoDock v4.2.6 software before docking, including removing water molecules, adding hydrogens and calculating the Gasteiger charge. The ligand was also preprocessed via AutoDock, including adding all hydrogens and detecting torsional bonds. The active pocket sites for small molecule ligand binding were obtained from the POCASA website (http://altair.sci.hokudai.ac.jp/g6/service/pocasa/). The pockets were sorted by volume depth, and the top five were picked for docking.^43^ AutoDock v4.2.6 software was used for docking, and the grid box was set to include the active pocket sites. Lamarck genetic Algorithm was selected and the number of docking was set to 50 times. Other docking parameters were set as default. Finally, the docking conformations were visualized by PyMOL v2.4.1 software.

### 5.7 Animals

Male BALB/c mice (22–25 g) aged 8–10 weeks were obtained from the animal center of Air Force Military Medical University of PLA (Xi'an, Shaanxi, China). All animal experiment protocols were performed in accordance with the guidelines of Animal Care and Use Committees at Northwest University (Xi'an, Shaanxi, China) and carried out according to the Guide for the Care and Use of Laboratory Animals published by the US National Institutes of Health (NIH Publication No. 85‐23, revised 2011). All mice had free access to food and water and were bred at 26°C in a 12 h light/12 h dark cycle.

### 5.8 Rodent model of sepsis

The CLP model was used to induce sepsis as previously described^44^ (Figure 4a,b). At 8 h post‐CLP, the murine sepsis score (MSS) and anal temperature were measured. The MSS system involves observing seven components: appearance, level of consciousness, activity, response to stimulus, eyes, respiratory rate, and respiratory quality. Each of these variables is given a score between 0 and 4.^25^ The established MSS score is the total of these seven components.

#### 5.8.1 Experimental design

MEL was dissolved in DMSO and given to mice every 2 days for 6 days before CLP injury. Mice were randomly assigned to the following groups: (a) mice injected with 1 ml/kg DMSO and subjected to sham surgery; (b) mice injected with 1 ml/kg DMSO and then subjected to CLP surgery; and (c) mice treated with MEL and then subjected to CLP surgery. In the third group, different levels of MEL (15, 30, or 60 mg/kg) were administrated to mice to monitor survival rate for 72 h, among which 30 mg/kg was selected for further analysis, including sepsis score, anal temperature, blood routine parameters, blood biochemical parameters, cardiac function, myocardial structure, and molecular signaling (Figure 4c).

### 5.9 Detection of blood routine parameters and blood biochemical parameters

At 8 h post‐CLP, at least 10 μl of blood was collected from the left eyeball into a heparin‐coated tube. The levels of WBC, LYM, MON, GRA, RBC, and PLT were detected by an automatic blood analyzer (Genrui Technology Co., Ltd, KT6200VET, Shenzhen, Guangdong, China). After that, 150 μl of serum was isolated from the rest of the whole blood by being centrifuged at 3000 rpm for 10 min. The levels of LDH, CK, AST, ALB, and BUN were detected by an automatic blood biochemical analyzer (XinRui Technology Co., Ltd, XR210, Zhongshan, Guangdong, China).

### 5.10 Echocardiography evaluation

Transthoracic echocardiography was performed using an animal‐specific instrument (VisualSonics Vevo3100, VisualSonics, Toronto, ON, Canada) at 8 h post‐CLP in mice. Mice were anesthetized with 3% isoflurane and 1 L/min 100% oxygen in an induction chamber for 1–2 min. Next, mice were, respectively, laid supine on a warm platform and kept anesthetized by 2% isoflurane until they lost body‐righting reflex. Then, series of M‐mode images at the level of papillary muscles were obtained. SV, CO, LVPWs, LVPWd, LVEDV, LVESV, and HR were measured using Vevo LAB 3.0.0. All measurements were based on three consecutive cardiac cycles.

### 5.11 Histological staining

The myocardium was fixed in 4% paraformaldehyde and sectioned at a thickness of 4–5 μm. Morphological changes in myocardium were observed by hematoxylin–eosin (H&E) staining. The degree of myocardial fibrosis was examined by Masson staining (Solarbio, Co., Ltd, Beijing, China). For immunostaining, paraffin‐embedded slices were stained with the respective primary antibody against NOX2, IL‐6, TNF‐α (1:200, Servicebio, Co., Ltd, Wuhan, Hubei, China), PIK3CG (1:200, Abclont Technology Co., Ltd, Boston, USA), then incubated with a secondary biotinylated anti‐rabbit IgG, stained with 3,3′‐diaminobenzidine (DAB), and imaged using a microscope (Invitrogen EVOS M5000, Thermo Fisher Scientific, Waltham, MA, USA). Finally, immunoreactive areas were quantified using the ImagePro Plus 4.5 software (Media Cybernetics, Silver Spring, USA).

### 5.12 Quantitative real‐time PCR

Total RNA was extracted from tissues using the TRIzolTM total RNA extraction kit (TAKARA BIO INC. Kusatsu, Shiga, Japan), and reverse transcription was performed using the Prime Script RT Master Mix (TAKARA BIO INC. Kusatsu, Shiga, Japan). Then TNF‐α, IL‐1β, IL‐6, NLRP3, PIK3CG, Akt, Myc, Pdk1, and Caspase‐1 mRNA level were detected using quantitative real‐time reverse transcriptase PCR analyses with SYBR Premix Ex Taq (Hunan Accurate Biotechnology Co. Ltd. Hunan, China). The primers that were used are shown in Table S1. The reaction conditions were as follows: (1) 95°C for 10 min, (2) 40 cycles of 95°C for 5 s and 60°C for 30 s, (3) 94°C for 30 s, 60°C for 90 s, 94°C for 10 s. The expression levels of the examined transcripts were compared to that of β‐actin and normalized to the mean value of the controls.

### 5.13 Cell culture, treatment, and establishment of the LPS‐induced cardiomyocyte injury model

HL‐1 cells were seeded and cultured as previously described.^45^ The specific steps used to establish LPS‐induced cardiomyocyte injury model were described below. First, HL‐1 cells seeded in 60 mm culture dishes were allowed to grow to approximately 80% confluence. Next, the medium was replaced with a fresh serum‐free medium. HL‐1 cells were treated with MEL (3 h) before exposure to LPS (3 h) to examine the effects of MEL against LPS‐induced cardiomyocyte injury. Finally, cell viability was detected using a Muse cell analyzer (Merck KGaA, Darmstadt, Germany).

### 5.14 Cell viability and intracellular ROS generation assessments

HL‐1 cells were seeded and cultured as previously described. The cell viability and intracellular ROS generation were examined according to our previous study.^45^

### 5.15 Western blotting

Isolated cells were homogenized in RIPA buffer containing protease and phosphatase inhibitors (Beyotime Biotechnology, Shanghai, China). Thirty‐fifty μg of total protein extract was loaded to SDS‐PAGE (8% or 10%) and transferred onto PVDF membranes (Millipore, Billerica, MA, USA), which were next blocked by 5% skim milk and incubated with primary antibodies at 4°C for 24 h. In this study, used primary antibodies were as follows: PIK3CG (1:1000, ABclonal Biotechnology Co.,Ltd, Wuhan, Hubei, China), AKT, p‐AKT, MYC, NLRP3 (1:1000, ProteinTech Group, Inc., Wuhan, Hubei, China) PDK1 (1:1000, Boster Biological Technology Co., Ltd, CA, USA), IL‐6 (1:‐500, Santa Cruz Biotechnology, Dallas, TX, USA), and β‐actin (1:1000, Servicebio, Wuhan, Hubei, China). Then, membranes were washed three times with TBST and incubated with secondary antibodies (Goat Anti‐mouse IgG, 1:2000, Biosharp, China; Goat Anti‐Rabbit IgG, 1:5000, Biosharp, China) at room temperature for 1 h. Subsequently, used the fluorescence signal to visualize the membrane and used the MiNiChemi610 imaging system for detection (Sagecreation Co., Ltd, Beijing, China). Finally, the signal was quantified by Image J 5.0 software (National Institutes of Health, Bethesda, MD, USA).

### 5.16 PIK3CG knockdown by siRNA

The sequences of siRNA targeting murine PIK3CG (sense, 5′‐CAAGAUCAGAGGCAUUGAUAUTT‐3′; antisense, 5′‐AUAUCAAUGCCUCUGAUCUUGTT‐3′) were synthesized by Sangon Biotechnology (Shanghai, China). The siRNA was used to knockdown PIK3CG in HL‐1 cells. The specific step can be referred to our previous studies.^45^

### 5.17 Statistical analysis

Data were analyzed using the GraphPad Prism 9.0.0 software (GraphPad Software Inc., San Diego, CA, USA). All values are presented as the mean ± standard deviation (SD). For analysis of experimental data, *t* test was used for comparison between two groups, and one‐way ANOVA was used for multiple group comparison. A *p* < 0.05 indicated significant differences.^46^

## AUTHOR CONTRIBUTIONS

**Qiong Liu:** Data curation (lead); formal analysis (lead); investigation (lead); methodology (lead); validation (equal); writing – original draft (lead); writing – review and editing (equal). **Yushu Dong:** Data curation (lead); investigation (lead); methodology (lead); writing – original draft (equal). **Germaine Escames:** Data curation (equal); investigation (lead); methodology (equal); writing – original draft (equal). **Xue Wu:** Investigation (equal); methodology (equal); writing – original draft (equal). **Jun Ren:** Investigation (equal); methodology (equal); writing – review and editing (equal). **Wenwen Yang:** Investigation (equal); methodology (equal). **Shaofei Zhang:** Investigation (equal); methodology (equal). **Yanli Zhu:** Investigation (equal); methodology (equal). **Ye Tian:** Investigation (equal); methodology (equal). **Yang Yang:** Conceptualization (lead); funding acquisition (lead); project administration (lead); resources (lead); supervision (lead); validation (lead); writing – review and editing (lead). **Darío Acuña‐Castroviejo:** Conceptualization (lead); investigation (equal); methodology (equal); project administration (equal); validation (lead); writing – original draft (equal); writing – review and editing (equal).

## FUNDING INFORMATION

This work was supported by the National Natural Science Foundation of China (81871607 and 82070422), Innovation Capability Strong Foundation Plan of Xi'an City (Medical Research Project, 21YXYJ0037), Key Research and Development Program of Shaanxi (2020ZDLSF04‐03), Major Research Projects of Xi'an Science and Technology Plan (201805104YX12SF38 (2)), and the Instituto de Salud Carlos III (Co‐funded by European Regional Development Fund/European Social Fund “Investing in your future”), Spain, grants PI19‐01372 and CB16/10/00239.

## CONFLICT OF INTERESTS

The authors declare no conflict of interest.

### PEER REVIEW

The peer review history for this article is available at https://publons.com/publon/10.1002/btm2.10384.

## Supporting information


**Appendix S1** Supporting Information

## Data Availability

The data that support the findings of this study are available from the corresponding author upon reasonable request.
