# Supplementary material for: Identification of PIK3CG as a hub in septic myocardial injury using network pharmacology and weighted gene co‐expression network analysis
Source: Bioeng Transl Med. 2022 Aug 3;8(1):e10384. doi: 10.1002/btm2.10384 (PMC9842026; doi:10.1002/btm2.10384)
Supplement: Supplementary file 1 — Appendix S1 Supporting Information [file BTM2-8-e10384-s001.docx]

**Supplementary Data**

**Identification of PIK3CG as a Hub in Septic Myocardial Injury using Network Pharmacology and Weighted Gene Co-expression Network Analysis**

Qiong Liu^#1, 2^, Yushu Dong^#3^, Germaine Escames^#4^, Xue Wu^1, 2^, Jun Ren^5, 6^, Wenwen Yang^1, 2^, Shaofei Zhang^1, 2^, Yanli Zhu^1, 2^, Ye Tian^1, 2^, Darío Acuña-Castroviejo^*4^, Yang Yang^*1, 2^

^1^Key Laboratory of Resource Biology and Biotechnology in Western China, Ministry of Education. Faculty of life Science and Medicine, Northwest University, Xi’an, China

^2^Xi’an Key Laboratory of Cardiovascular and Cerebrovascular Diseases, Xi’an No.3 Hospital, The Affiliated Hospital of Northwest University. Faculty of life science and Medicine, Northwest University, Xi’an, China

^3^Institute of Neuroscience, General Hospital of Northern Theater Command, Shenyang, China

^4^Biomedical Research Center, Health Sciences Technology Park, University of Granada, Avda. del Conocimiento s/n; Granada, Spain; Ibs. Granada and CIBERfes, Granada, Spain; and UGC of Clinical Laboratories, Universitu San Cecilio's Hospital, Granada, Spain.

^5^Department of Cardiology, Zhongshan Hospital, Fudan University; Shanghai Institute of Cardiovascular Diseases, Shanghai, China

^6^Department of Laboratory Medicine and Pathology, University of Washington, Seattle, WA, USA

^#^These authors contributed equally to this work.

**^*^Address correspondence to:**

Yang Yang MD., PhD. and Darío Acuña-Castroviejo, MD., PhD.

Key Laboratory of Resource Biology and Biotechnology in Western China, Ministry of Education

Faculty of life science and Medicine, Northwest University

229 Taibai North Road

Xi’an 710069, China

Telephone: +86 13379217366

Email address: [yang200214yy@nwu.edu.cn](mailto:yang200214yy@nwu.edu.cn) (Yang Yang) and dacuna@ugr.es (Darío Acuña-Castroviejo)

**Supplemental Figures**

**
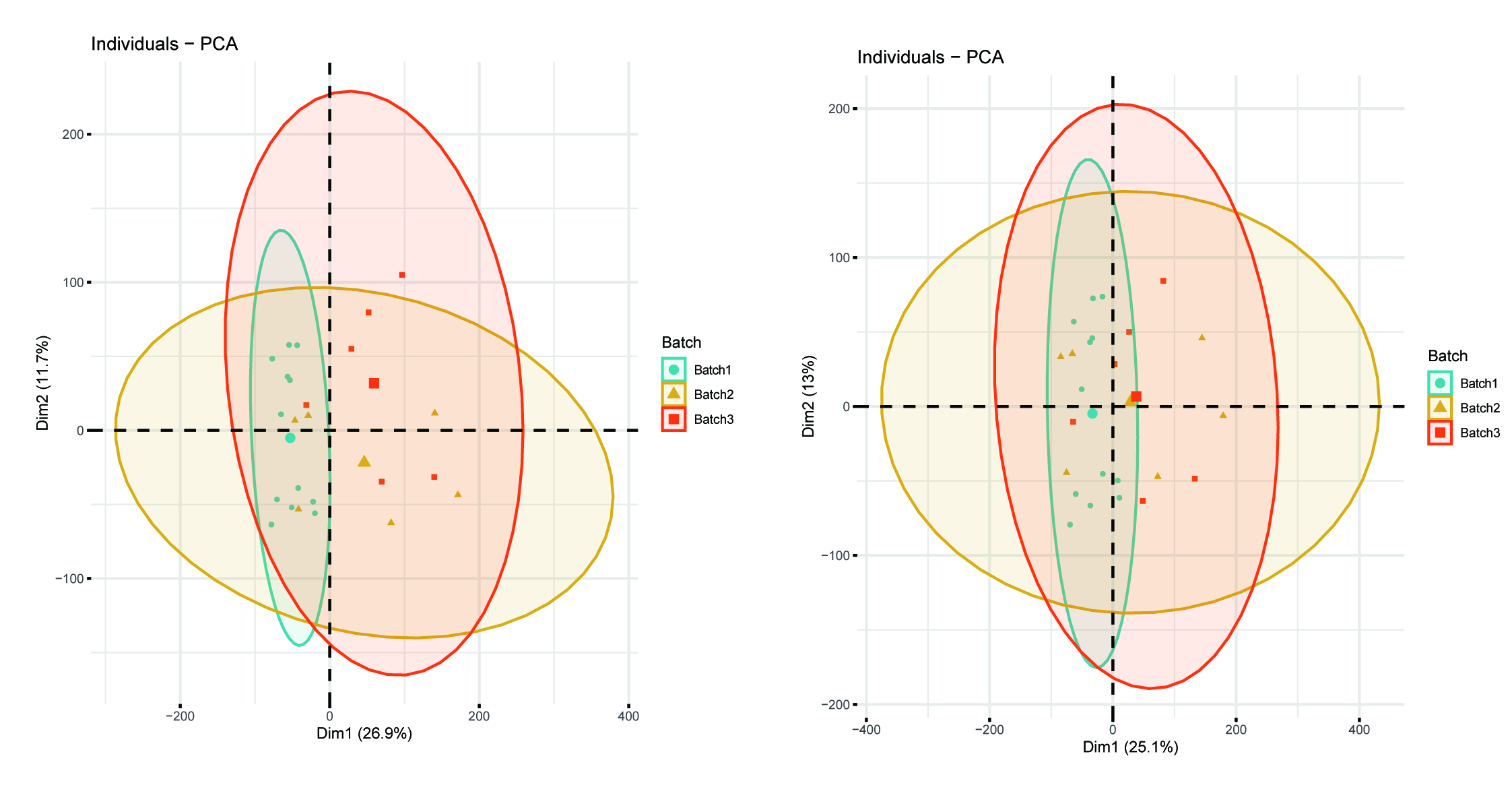
**

**Figure S1.** Principal component diagrams visualizing protein expression profiles. Samples (batch 1, 2, 3; n=6, 6, 6) are plotted along the first two principal component axes (PC1 and PC2) before (left) and after (right) batch effect correction.


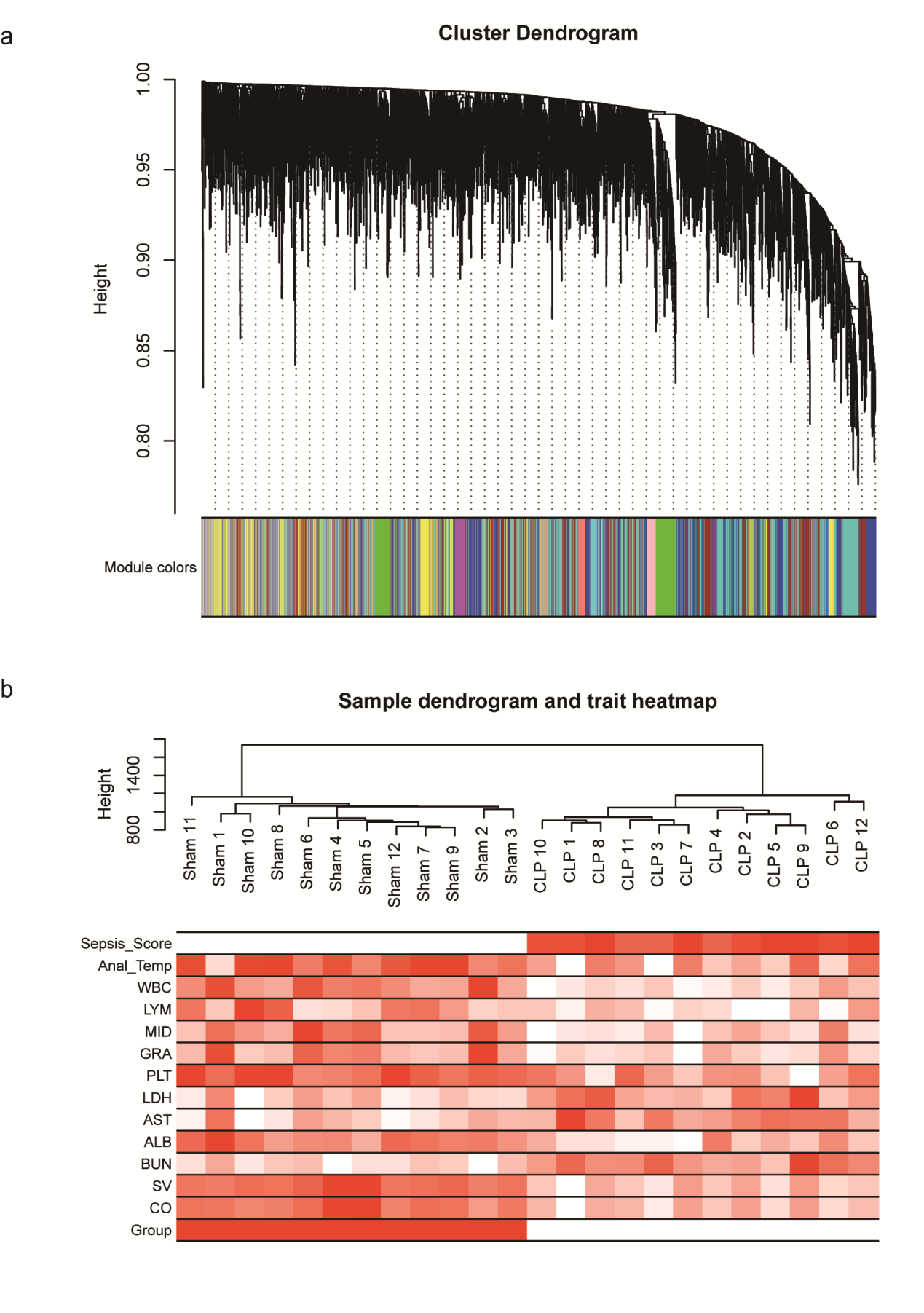


**Figure S2.** Hierarchical clustering dendrograms of WGCNA. **(a)** Hierarchical clustering dendrograms of identified co-expressed genes in modules. Totaling nine coexpression modules are constructed and exhibited in different colors. **(b)** The hierarchical clustering dendrogram of all samples and the corresponding sepsis features heatmap. The gradient from dark red to white in the heatmap represents the high to low level of the sepsis characters respectively. WGCNA, weighted gene co-expression network analysis.

**Table S1.** Sequences of primers used in PCR amplification.

| Gene | Primer | Sequence |
| --- | --- | --- |
| NLRP3 | Forward | ACCTCAACAGTCGCTACAC |
|  | Reverse | GTCCTCGGGCTCAAACA |
| PDK1 | Forward | TGCACAGTACTTCCAGGGAG |
|  | Reverse | TGCACAGTACTTCCAGGGAG |
| Akt | Forward | CTGCCCTTCTACAACCAGGA |
|  | Reverse | CATACACATCCTGCCACACG |
| IL-6 | Forward | TACCACTTCACAAGTCGGAGGC |
|  | Reverse | CTGCAAGTGCATCATCGTTGTT |
| TNF-α | Forward | GGTGCCTATGTCTCAGCCTCTT |
|  | Reverse | GCCATAGAACTGATGAGAGGGAG |
| Myc | Forward | GGACTGTATGTGGAGCGGTTTC |
|  | Reverse | TCGTTGAGCGGGTAGGGA |
| IL-1β | Forward | TGGACCTTCCAGGATGAGGACA |
|  | Reverse | GTTCATCTCGGAGCCTGTAGTG |
| Caspase-1 | Forward | AGAACAGAACAAAGAAGATGGCACA |
|  | Reverse | GTGCCATCTTCTTTGTTCTGTTCTT |
| PIK3CG | Forward | CCTGTGCTGAAACTGTGCTT |
|  | Reverse | TGTGGTGTCGGAAGCTAAGT |

**Table S2.** List of sepsis-related genes collected from known databases and current analysis.

|  | Terms | Gene |
| --- | --- | --- |
| Known databases | TCMIP  (77 genes) | ABCA12,ACTG2,ADA,AK2,ALOX12B,ALOXE3,APC,ATP7A,BLNK,BTK,CD79A,CD79B,CHD7,CTNNB1,CYBA,CYBB,CYP4F22,DCLRE1C,ECE1,EDN3,EDNRB,ELANE,FERMT3,G6PC3,GALT,GDNF,HLA-B,IGHM,IGLL1,IKZF1,IL2RG,IL7R,ITGB4,LIG4,LIPN,LMOD1,LRRC8A,MNX1,MUT,MYH11,MYLK,NCF1,NCF2,NCF4,NFKB2,NIPAL4,NRTN,PIK3R1,PLEC,RAG1,RAG2,RET,RMRP,SAMD9,SDR9C7,SEMA3C,SEMA3D,SULT2B1,TCF3,TFRC,TGM1,WAS,WIPF1,IRAK4,NOS3,MIF,BDKRB2,F5,F8,CD14,F3,CTSC,MYD88,F11,TFPI,TNF,PIK3CG |
|  | TCMSP  (5 genes) | F5,NOS3,MIF,BDKRB2,IRAK4 |
|  | DisGeNET  (37 genes) | TNF,TLR4,HMGB1,IL10,ADM,IFNG,PROC,NOS2,CASP3,MMP9,CSF3,ANGPT1,LTA,LBP,MAPK1,MIF,TGFB1,NOS3,TGFBI,CXCR2,IL6,CRP,IL1B,TLR2,F3,CCL2,THBD,STAT3,SERPINE1,IL17A,AGER,IL1RN,ICAM1,IFNA2,MAPK3,ADORA2B,ADORA2A |
| Transcriptional analysis | WGCNA  (156 genes) | UNC5B,SERTAD4,LRCH1,CD55,KLHL4,GULP1,MRM1,CHEK2,WNT5A,ATP1B2,STON2,MAPK6,CYP2J2,CRY1,SYBU,COLEC11,MS4A8,KCNK2,DOC2B,NHSL2,HIF3A,GJA5,DCTN5,PSEN2,ACR,ZC3H8,PAQR9,PCIF1,MYO6,CA8,LDLRAD4,EIF2AK4,ITIH4,PRPF38A,SCGB3A1,SOAT2,SLCO2A1,TNFAIP6,FST,SLC19A2,NET1,SOWAHC,SSH3,NEK6,STXBP2,TWF1,SLC9A3R1,DOK3,SH2B2,GMFG,CCL3L1,CNTFR,MPZL2,ASCC2,CACNA1D,LILRB1,HS6ST1,BST1,SAMSN1,RAB4B-EGLN2,SEMA3A,KCNN3,WFIKKN2,MARCKSL1,ZSWIM4,ADAMTS14,COL13A1,SAA1,NMRAL1,ASPG,SIGLEC8,IGSF6,TBRG1,ORM2,MCTP2,ORAI2,PROM1,CFAP69,ADGRG3,AOAH,LRRC25,SERPINA3,VPS11,OSR1,PAWR,SECTM1,A4GALT,IFITM3,BMPER,IL18RAP,ACAT2,PAPPA,ACKR1,TRAF6,MAMSTR,EPHA2,ACP5,ITIH3,SH2D5,GAN,MTMR12,OR5M3,ITPKC,LCP2,NUMBL,GLIS3,MRPL52,CLEC4C,CLEC5A,LPCAT2,NLRP3,SLC9A1,FHL3,DCXR,ADM,TNFSF11,KRI1,IL17RA,DUSP2,CDC42SE1,SLC6A12,SPTBN5,PGM1,FJX1,TMEM119,SCN3A,NRIP1,SECISBP2L,SPHKAP,FLT3LG,CACNA1A,KCNA4,SH2D4A,FGF9,FAM219B,DACH1,RBBP5,IGIP,KATNAL1,PHLPP1,ACKR4,RDH13,PPP1R26,ADAL,SPICE1,PEX26,SMTNL2,MTCL1,ABCA12,TMEM74B,XRCC2,MAPKAPK3,TMTC2,EEPD1,PIGO,TRHDE |
|  | RNA-seq (Top 200 DEGs) | CSF3,SAA3,SAA2,SAA1,ORM1,NGP,FGA,GPR84,TNFSF11,CXCL2,LOC101056219,IFITM7,GM32089,LOC108168045,STFA2L1,AA467197,FPR1,GRK1,CCL19-PS3,PRSS16,CHIL1,GM32296,CXCL13,ANKRD22,LIPG,ACOD1,GM15987,UNC5CL,LCN2,CLEC4E,FPR2,FGG,AQP3,TMEM252,CCL17,FAM196A,GM36753,TMEM8C,ADAMTS4,SERPINA3M,GM29994,IL1RN,GM39291,LOC108168962,GM42023,SERPINA3N,HP,PGLYRP3,WFDC21,D330045A20RIK,CCL3,RPGRIP1,GM10851,PSG22,CFB,ORM3,PTX3,SERPINA3C,FCRLB,GM9696,AI463229,CPNE9,CXCL1,CAMP,GM32925,MS4A8A,LOC108168952,IL10,FAM110C,LOC108167926,AKAP2,SH2D5,APOA1,MRGPRA2B,FFAR4,FCGR4,CNTFR,SLC6A12,3930402G23RIK,GM38528,GM5841,LDLRAD1,LOC108169150,CXCL14,AOC1,CLCA3A1,IL1B,GM42221,INHBB,SEC14L4,GM38407,SECTM1A,PRG2,OLFM4,GM40960,LBP,FGFR4,ACAT3,GM34907,ADORA3,TMIGD1,GRIN3A,CYP2S1,FGD2,LDHAL6B,ITGB6,GM4793,SOX9,LOC105243866,GM10451,GM42012,ESM1,GM32849,AIP,CLEC4G,E030013I19RIK,2610036A22RIK,GM31735,CD180,SLC15A2,GM18706,SLC39A2,AQP8,6430584L05RIK,GM26708,MYCN,GM11816,LOC108168167,RMI2,CD300LD4,FAM198A,F2RL1,GM20162,FRMD7,LAIR1,TLR11,1810006J02RIK,BANK1,CCL24,AI427809,GM15867,GM36569,GM34140,GALNT12,GM41640,GM17853,GM9844,GM31563,GM35083,GM19673,LRRC55,2610035D17RIK,APLNR,GM26691,S100A7A,PCYT1B,LRRC17,BLNK,SPON2,PIEZO2,GM18113,SYT17,GM39444,GM32918,AASS,AKAP5,LDOC1,GM26901,DMRTA2,GPR34,GM27151,GM24175,ST8SIA2,LRRC15,MARCKSL1-PS4,GM39285,TENM2,TNFRSF17,ATP6V0A4,NAT8F3,LYPD1,LOC108167554,TSPAN32,NECAB1,TLL2,SLC27A6,CD300LD3,FBXL13,GM31633,ABCG3,GM13749,GM40080,ARMC3,CX3CR1,PRRX2,LOC433198,GM12426,CLEC7A,B3GALT2,GM2115 |
| Merged targets  (453 genes) | | ABCA12,ACTG2,ADA,AK2,ALOX12B,ALOXE3,APC,ATP7A,BLNK,BTK,CD79A,CD79B,CHD7,CTNNB1,CYBA,CYBB,CYP4F22,DCLRE1C,ECE1,EDN3,EDNRB,ELANE,FERMT3,G6PC3,GALT,GDNF,HLA-B,IGHM,IGLL1,IKZF1,IL2RG,IL7R,ITGB4,LIG4,LIPN,LMOD1,LRRC8A,MNX1,MUT,MYH11,MYLK,NCF1,NCF2,NCF4,NFKB2,NIPAL4,NRTN,PIK3R1,PLEC,RAG1,RAG2,RET,RMRP,SAMD9,SDR9C7,SEMA3C,SEMA3D,SULT2B1,TCF3,TFRC,TGM1,WAS,WIPF1,IRAK4,NOS3,MIF,BDKRB2,F5,F8,CD14,F3,CTSC,MYD88,F11,TFPI,TNF,PIK3CG,TLR4,HMGB1,IL10,ADM,IFNG,PROC,NOS2,CASP3,MMP9,CSF3,ANGPT1,LTA,LBP,MAPK1,TGFB1,TGFBI,CXCR2,IL6,CRP,IL1B,TLR2,CCL2,THBD,STAT3,SERPINE1,IL17A,AGER,IL1RN,ICAM1,IFNA2,MAPK3,ADORA2B,ADORA2A,UNC5B,SERTAD4,LRCH1,CD55,KLHL4,GULP1,MRM1,CHEK2,WNT5A,ATP1B2,STON2,MAPK6,CYP2J2,CRY1,SYBU,COLEC11,MS4A8,KCNK2,DOC2B,NHSL2,HIF3A,GJA5,DCTN5,PSEN2,ACR,ZC3H8,PAQR9,PCIF1,MYO6,CA8,LDLRAD4,EIF2AK4,ITIH4,PRPF38A,SCGB3A1,SOAT2,SLCO2A1,TNFAIP6,FST,SLC19A2,NET1,SOWAHC,SSH3,NEK6,STXBP2,TWF1,SLC9A3R1,DOK3,SH2B2,GMFG,CCL3L1,CNTFR,MPZL2,ASCC2,CACNA1D,LILRB1,HS6ST1,BST1,SAMSN1,RAB4B-EGLN2,SEMA3A,KCNN3,WFIKKN2,MARCKSL1,ZSWIM4,ADAMTS14,COL13A1,SAA1,NMRAL1,ASPG,SIGLEC8,IGSF6,TBRG1,ORM2,MCTP2,ORAI2,PROM1,CFAP69,ADGRG3,AOAH,LRRC25,SERPINA3,VPS11,OSR1,PAWR,SECTM1,A4GALT,IFITM3,BMPER,IL18RAP,ACAT2,PAPPA,ACKR1,TRAF6,MAMSTR,EPHA2,ACP5,ITIH3,SH2D5,GAN,MTMR12,OR5M3,ITPKC,LCP2,NUMBL,GLIS3,MRPL52,CLEC4C,CLEC5A,LPCAT2,NLRP3,SLC9A1,FHL3,DCXR,TNFSF11,KRI1,IL17RA,DUSP2,CDC42SE1,SLC6A12,SPTBN5,PGM1,FJX1,TMEM119,SCN3A,NRIP1,SECISBP2L,SPHKAP,FLT3LG,CACNA1A,KCNA4,SH2D4A,FGF9,FAM219B,DACH1,RBBP5,IGIP,KATNAL1,PHLPP1,ACKR4,RDH13,PPP1R26,ADAL,SPICE1,PEX26,SMTNL2,MTCL1,TMEM74B,XRCC2,MAPKAPK3,TMTC2,EEPD1,PIGO,TRHDE,SAA3,SAA2,ORM1,NGP,FGA,GPR84,CXCL2,LOC101056219,IFITM7,GM32089,LOC108168045,STFA2L1,AA467197,FPR1,GRK1,CCL19-PS3,PRSS16,CHIL1,GM32296,CXCL13,ANKRD22,LIPG,ACOD1,GM15987,UNC5CL,LCN2,CLEC4E,FPR2,FGG,AQP3,TMEM252,CCL17,FAM196A,GM36753,TMEM8C,ADAMTS4,SERPINA3M,GM29994,GM39291,LOC108168962,GM42023,SERPINA3N,HP,PGLYRP3,WFDC21,D330045A20RIK,CCL3,RPGRIP1,GM10851,PSG22,CFB,ORM3,PTX3,SERPINA3C,FCRLB,GM9696,AI463229,CPNE9,CXCL1,CAMP,GM32925,MS4A8A,LOC108168952,FAM110C,LOC108167926,AKAP2,APOA1,MRGPRA2B,FFAR4,FCGR4,3930402G23RIK,GM38528,GM5841,LDLRAD1,LOC108169150,CXCL14,AOC1,CLCA3A1,GM42221,INHBB,SEC14L4,GM38407,SECTM1A,PRG2,OLFM4,GM40960,FGFR4,ACAT3,GM34907,ADORA3,TMIGD1,GRIN3A,CYP2S1,FGD2,LDHAL6B,ITGB6,GM4793,SOX9,LOC105243866,GM10451,GM42012,ESM1,GM32849,AIP,CLEC4G,E030013I19RIK,2610036A22RIK,GM31735,CD180,SLC15A2,GM18706,SLC39A2,AQP8,6430584L05RIK,GM26708,MYCN,GM11816,LOC108168167,RMI2,CD300LD4,FAM198A,F2RL1,GM20162,FRMD7,LAIR1,TLR11,1810006J02RIK,BANK1,CCL24,AI427809,GM15867,GM36569,GM34140,GALNT12,GM41640,GM17853,GM9844,GM31563,GM35083,GM19673,LRRC55,2610035D17RIK,APLNR,GM26691,S100A7A,PCYT1B,LRRC17,SPON2,PIEZO2,GM18113,SYT17,GM39444,GM32918,AASS,AKAP5,LDOC1,GM26901,DMRTA2,GPR34,GM27151,GM24175,ST8SIA2,LRRC15,MARCKSL1-PS4,GM39285,TENM2,TNFRSF17,ATP6V0A4,NAT8F3,LYPD1,LOC108167554,TSPAN32,NECAB1,TLL2,SLC27A6,CD300LD3,FBXL13,GM31633,ABCG3,GM13749,GM40080,ARMC3,CX3CR1,PRRX2,LOC433198,GM12426,CLEC7A,B3GALT2,GM2115 |

**Table S3.** List of MEL targets predicted via Drugbank database.

| Terms | Gene |
| --- | --- |
| MEL targets (Drugbank similarity threshold > 0.6; 109 genes) | PDPK1;HTR1A;HTR1B;HTR1D;HTR1F;HTR2A;HTR2B;HTR2C;HTR3A;HTR3B;HTR4;ASMT;ACTR2;ARPC1B;ARPC2;ARPC3;ARPC4;ARPC5;ACTR3;ADA;ADRA1A;ADRA1B;ADRA1D;ADRA2A;ADRA2B;ADRA2C;ADRB1;ADRB2;ADRB3;CALM1;CALR;CASP1;CTSS;chiB;F10;CDK2;DRD2;DRD3;DRD4;DAPK1;SELE;EPX;ESR1;FGFR1;GJA1;QPCT;GSK3B;HNMT;EZH2;HIF1A;KCNJ4;KRT7;LTA4H;MAPKAPK2;MAPKAPK3;MMP7;MTNR1A;MTNR1B;MAPK14;MAP3K9;CHRM1;MPO;NDUFC2;NPPB;CHRNA4;CHRNB2;RORB;PPARG;PIK3CG;PLA2G2A;SERPINA5;PARP1;PARP2;PARP3;KCNH2;PHB2;PRKCQ;tgt;NQO2;SKP1;SPR;PIM1;SIGMAR1;SLC6A4;SCN10A;SCN5A;SLC18A2;TRPV1;trpA;trpB;CSK;ITK;LCK;SYK;ZAP70;VCAM1;VEGFA;KDR;WEE1;PDE4A;PDE4B;PDE4D;CYP1A1;CYP1A2;CYP1B1;CYP2C19;CYP2C9;IDO1;CYP19A1; |

**Table S4.** Mice biometric, plasma and echocardiographic parameters

| Mice | Sepsis score | Anal_  Temperature | WBC | LYM | MID | GRA | RBC | PLT | LDH | AST | ALB | BUN | SV | CO | Group |
| --- | --- | --- | --- | --- | --- | --- | --- | --- | --- | --- | --- | --- | --- | --- | --- |
| CLP 1 | 8 | 28.7 | 2.9 | 0.452 | 0.52 | 2.38 | 13.37 | 441.5 | 1718.8 | 568.2 | 20.8 | 11.8 | 5.55783 | 1.667349 | 1 |
| CLP 2 | 8 | 32.5 | 3.9 | 0.235 | 1.29 | 2.375 | 11.025 | 455 | 1672.4 | 365.8 | 21.5 | 8 | 21.28787 | 8.30227 | 1 |
| CLP 3 | 7 | 28.5 | 4.05 | 0.325 | 1.02 | 2.55 | 11.97 | 417.5 | 1072.8 | 393.8 | 20.2 | 12 | 9.134831 | 2.932281 | 1 |
| CLP 4 | 7 | 30.8 | 2.85 | 0.78 | 0.915 | 3.535 | 12.135 | 446.5 | 950.9 | 287.7 | 25.5 | 7.7 | 14.09241 | 5.284655 | 1 |
| CLP 5 | 9 | 30.6 | 2.75 | 0.215 | 0.87 | 1.66 | 11.79 | 338 | 1530.6 | 420 | 22.9 | 6.4 | 10.1704 | 3.895265 | 1 |
| CLP 6 | 8 | 30.1 | 6.2 | 0.375 | 1.88 | 4.32 | 11.85 | 412 | 1024.4 | 399.8 | 24.5 | 11.2 | 12.10561 | 4.031168 | 1 |
| CLP 7 | 9 | 34.8 | 2.3 | 1 | 0.3 | 1 | 11.83 | 328 | 1157.3 | 268.2 | 19.8 | 8.6 | 22.76792 | 8.333059 | 1 |
| CLP 8 | 9 | 34.9 | 3.7 | 1.6 | 0.6 | 1.8 | 12.75 | 264 | 1888.7 | 398.1 | 20.8 | 9 | 19.7277 | 7.318976 | 1 |
| CLP 9 | 9 | 36.3 | 4 | 1.5 | 0.6 | 2 | 11.64 | 232 | 2181.8 | 402.8 | 21.7 | 14.3 | 23.07471 | 8.883763 | 1 |
| CLP 10 | 8 | 32.6 | 2.1 | 1.2 | 0.3 | 1.2 | 12.49 | 535 | 1339 | 239.3 | 22.9 | 8.4 | 14.08357 | 6.872781 | 1 |
| CLP 11 | 7 | 33.3 | 3.1 | 1.4 | 0.5 | 2.1 | 14.55 | 577 | 1225.4 | 203.7 | 20.2 | 9.2 | 14.07093 | 6.585195 | 1 |
| CLP 12 | 9 | 35.7 | 4.4 | 2.2 | 0.6 | 2.1 | 14.32 | 540 | 1332.2 | 243.1 | 21.8 | 9.4 | 14.33863 | 6.15127 | 1 |
| Sham 1 | 0 | 29.8 | 10.85 | 1.145 | 2.17 | 7.53 | 13.205 | 555 | 1484.4 | 413.6 | 29 | 7.6 | 31.13332 | 12.994862 | 2 |
| Sham 2 | 0 | 34.2 | 11.75 | 0.985 | 2.565 | 8.2 | 10.61 | 596 | 912.5 | 262.9 | 25.5 | 4.1 | 30.26316 | 9.290789 | 2 |
| Sham 3 | 0 | 35.7 | 5.55 | 1.14 | 1.44 | 3.465 | 9.97 | 564 | 887.1 | 230.6 | 24.2 | 6.6 | 33.15061 | 12.66353 | 2 |
| Sham 4 | 0 | 37.8 | 7.8 | 0.81 | 2.04 | 4.945 | 10.135 | 490.5 | 1016.7 | 232 | 24.6 | 4 | 47.08867 | 18.36458 | 2 |
| Sham 5 | 0 | 34.4 | 8.4 | 1.23 | 2.35 | 5.32 | 9.81 | 545 | 1248.1 | 186.7 | 22.9 | 5.2 | 46.03195 | 19.05723 | 2 |
| Sham 6 | 0 | 35.1 | 10.25 | 0.675 | 3.015 | 6.56 | 10.125 | 477.5 | 1287.1 | 282.6 | 24.5 | 6.5 | 37.32581 | 14.18381 | 2 |
| Sham 7 | 0 | 38.8 | 5.4 | 3.3 | 0.9 | 2.5 | 11.97 | 593 | 848.5 | 140.5 | 25.9 | 6.3 | 34.19908 | 13.91902 | 2 |
| Sham 8 | 0 | 38.9 | 5.4 | 3.8 | 1.2 | 2.8 | 11.69 | 687 | 966.8 | 145.1 | 23.6 | 6.1 | 33.12383 | 11.82521 | 2 |
| Sham 9 | 0 | 39.2 | 5.6 | 2.2 | 1 | 2.8 | 12.17 | 535 | 1068.9 | 189.7 | 25.1 | 6 | 36.40861 | 13.43478 | 2 |
| Sham 10 | 0 | 38.6 | 6.2 | 4.8 | 1.5 | 2.4 | 12.6 | 676 | 677.9 | 99.8 | 26 | 4.9 | 34.79456 | 11.31175 | 2 |
| Sham 11 | 0 | 38.3 | 7 | 3.2 | 0.9 | 2.9 | 10.96 | 693 | 786.2 | 121.6 | 26.6 | 5.1 | 32.17042 | 13.44724 | 2 |
| Sham 12 | 0 | 37.5 | 7.1 | 3.1 | 0.9 | 3 | 11.29 | 690 | 758.9 | 113 | 26.6 | 5.2 | 32.86434 | 11.44326 | 2 |

**Note:** WBC, white blood cells, LYM, lymphocytes, MID, middle cells, GRA, granulocytes, and PLT, platelets, RBC, red blood cells, LDH, lactic dehydrogenase, CK, creatine kinase, BUN, blood urea nitrogen, AST, aspartate aminotransferase, SV, stroke volume, CO, cardiac output

**Table S5.** Hub gene list generated from the pharmacological network analysis.

| Node | Hub gene | Degree | Closeness | Betweenness |
| --- | --- | --- | --- | --- |
| IL6 | TRUE | 26 | 0.06 | 1610.161 |
| PIK3CG | TRUE | 25 | 0.059 | 1112.766 |
| BDKRB2 | TRUE | 21 | 0.059 | 615.487 |
| IL10 | TRUE | 21 | 0.06 | 1089.723 |
| CXCL1 | TRUE | 21 | 0.06 | 1288.441 |
| CXCR2 | TRUE | 20 | 0.059 | 638.667 |
| CD14 | TRUE | 20 | 0.059 | 1257.463 |
| CCL2 | TRUE | 19 | 0.06 | 1042.228 |
| TRAF6 | TRUE | 19 | 0.059 | 1582.774 |
| ADRA2A | TRUE | 17 | 0.058 | 164.578 |
| PIK3R1 | TRUE | 17 | 0.059 | 2502.372 |
| ADRA2B | TRUE | 16 | 0.058 | 76.311 |
| DRD2 | TRUE | 16 | 0.058 | 57.423 |
| TLR4 | TRUE | 16 | 0.059 | 788.563 |
| APLNR | TRUE | 15 | 0.058 | 421.55 |
| ADORA3 | TRUE | 15 | 0.058 | 50.677 |
| CCL3 | TRUE | 15 | 0.059 | 1018.434 |
| HTR2C | TRUE | 14 | 0.058 | 126.952 |
| MAPK3 | TRUE | 14 | 0.06 | 1826.815 |
| ICAM1 | TRUE | 14 | 0.059 | 1315.043 |
| HTR2B | TRUE | 13 | 0.058 | 59.793 |
| IFNG | TRUE | 13 | 0.059 | 195.389 |
| TLR2 | TRUE | 12 | 0.059 | 330.453 |
| ADRA1B | TRUE | 11 | 0.057 | 178.509 |
| ADRA1A | TRUE | 11 | 0.057 | 178.509 |
| MYD88 | TRUE | 11 | 0.058 | 130.374 |
| ADRB1 | TRUE | 10 | 0.059 | 610.311 |
| WAS | TRUE | 10 | 0.057 | 875.669 |
| MAPK1 | TRUE | 10 | 0.058 | 670.822 |
| ADORA2A | TRUE | 9 | 0.058 | 270.314 |
| CASP3 | TRUE | 8 | 0.058 | 486.497 |
| IL7R | TRUE | 8 | 0.058 | 464.861 |
| IL17A | TRUE | 8 | 0.059 | 53.647 |
| TNFSF11 | TRUE | 8 | 0.059 | 532.631 |
| HLA-B | TRUE | 7 | 0.057 | 217.353 |
| CTNNB1 | TRUE | 7 | 0.057 | 223.344 |
| IL1B | TRUE | 7 | 0.058 | 107.485 |
| IL2RG | TRUE | 7 | 0.058 | 177.722 |
| TGFB1 | TRUE | 7 | 0.058 | 426.576 |
| STAT3 | TRUE | 7 | 0.059 | 680.945 |
| ARPC2 | TRUE | 7 | 0.058 | 222.786 |
| ACTR2 | TRUE | 7 | 0.057 | 196.448 |
| LILRB1 | TRUE | 7 | 0.057 | 249.627 |
| SERPINE1 | TRUE | 7 | 0.056 | 708.207 |
| MMP9 | TRUE | 6 | 0.059 | 775.974 |
| RET | TRUE | 6 | 0.058 | 783.889 |
| BTK | TRUE | 6 | 0.056 | 696.134 |
| LBP | TRUE | 6 | 0.058 | 483.982 |
| ADM | TRUE | 6 | 0.058 | 158.387 |
| LILRB2 | TRUE | 6 | 0.057 | 278.179 |
| THBD | TRUE | 5 | 0.057 | 593.218 |
| MYLK | TRUE | 5 | 0.058 | 1255.867 |
| NFKB2 | TRUE | 5 | 0.058 | 192.708 |
| TFRC | TRUE | 5 | 0.058 | 158.755 |
| CHEK2 | TRUE | 5 | 0.057 | 735.2 |
| LILRB3 | TRUE | 5 | 0.056 | 52.646 |
| PGM1 | TRUE | 5 | 0.056 | 697.13 |
| MYH11 | TRUE | 4 | 0.056 | 73.61 |
| ACTG2 | TRUE | 4 | 0.056 | 815 |
| APOA1 | TRUE | 4 | 0.056 | 498.316 |
| FPR1 | TRUE | 4 | 0.057 | 825.671 |
| IL17RA | TRUE | 4 | 0.058 | 107.877 |
| LCP2 | TRUE | 4 | 0.057 | 264.297 |
| TCF3 | TRUE | 4 | 0.057 | 192.373 |
| PSEN2 | TRUE | 4 | 0.057 | 89.849 |
| ITIH4 | TRUE | 4 | 0.057 | 978.872 |

**Table S6.** GO term enrichment of the Hub genes.

| ID | Description | ONTOLOGY | Count | GeneRatio | BgRatio | Pvalue | p.adjust | qvalue |
| --- | --- | --- | --- | --- | --- | --- | --- | --- |
| GO:0032496 | Response to lipopolysaccharide | BP | 23 | 23/66 | 326/18862 | 2.39E-24 | 6.92E-21 | 3.15E-21 |
| GO:0002237 | Response to molecule of bacterial origin | BP | 23 | 23/66 | 346/18862 | 9.42E-24 | 1.36E-20 | 6.21E-21 |
| GO:0071216 | Cellular response to biotic stimulus | BP | 20 | 20/66 | 233/18862 | 7.38E-23 | 5.64E-20 | 2.57E-20 |
| GO:0001819 | Positive regulation of cytokine production | BP | 24 | 24/66 | 437/18862 | 7.80E-23 | 5.64E-20 | 2.57E-20 |
| GO:0071222 | Cellular response to lipopolysaccharide | BP | 19 | 19/66 | 197/18862 | 1.06E-22 | 6.17E-20 | 2.81E-20 |
| GO:0071219 | Cellular response to molecule of bacterial origin | BP | 19 | 19/66 | 209/18862 | 3.36E-22 | 1.62E-19 | 7.37E-20 |
| GO:0031663 | Lipopolysaccharide-mediated signaling pathway | BP | 12 | 12/66 | 58/18862 | 9.21E-19 | 3.81E-16 | 1.73E-16 |
| GO:0032755 | Positive regulation of interleukin-6 production | BP | 12 | 12/66 | 86/18862 | 1.47E-16 | 5.31E-14 | 2.42E-14 |
| GO:0032675 | Regulation of interleukin-6 production | BP | 14 | 14/66 | 158/18862 | 2.48E-16 | 7.98E-14 | 3.63E-14 |
| GO:0032635 | Interleukin-6 production | BP | 14 | 14/66 | 162/18862 | 3.54E-16 | 1.02E-13 | 4.66E-14 |
| GO:0045121 | Membrane raft | CC | 12 | 12/66 | 323/19520 | 7.61E-10 | 7.68E-08 | 5.41E-08 |
| GO:0098857 | Membrane microdomain | CC | 12 | 12/66 | 323/19520 | 7.61E-10 | 7.68E-08 | 5.41E-08 |
| GO:0009897 | External side of plasma membrane | CC | 10 | 10/66 | 402/19520 | 9.26E-07 | 6.24E-05 | 4.39E-05 |
| GO:0044853 | Plasma membrane raft | CC | 5 | 5/66 | 111/19520 | 3.68E-05 | 0.001332724 | 0.00093756 |
| GO:0005769 | Early endosome | CC | 8 | 8/66 | 378/19520 | 3.96E-05 | 0.001332724 | 0.00093756 |
| GO:0030667 | Secretory granule membrane | CC | 7 | 7/66 | 305/19520 | 7.51E-05 | 0.002167045 | 0.001524497 |
| GO:0034774 | Secretory granule lumen | CC | 7 | 7/66 | 322/19520 | 0.00010533 | 0.002385912 | 0.001678468 |
| GO:0060205 | Cytoplasmic vesicle lumen | CC | 7 | 7/66 | 326/19520 | 0.00011372 | 0.002385912 | 0.001678468 |
| GO:0031983 | Vesicle lumen | CC | 7 | 7/66 | 328/19520 | 0.000118114 | 0.002385912 | 0.001678468 |
| GO:0072562 | Blood microparticle | CC | 5 | 5/66 | 146/19520 | 0.000135324 | 0.002485039 | 0.001748204 |
| GO:0005126 | Cytokine receptor binding | MF | 13 | 13/66 | 270/18337 | 1.17E-11 | 2.78E-09 | 1.85E-09 |
| GO:0008227 | G protein-coupled amine receptor activity | MF | 7 | 7/66 | 51/18337 | 5.76E-10 | 6.86E-08 | 4.55E-08 |
| GO:0005125 | Cytokine activity | MF | 10 | 10/66 | 235/18337 | 1.11E-08 | 8.82E-07 | 5.85E-07 |
| GO:0048018 | Receptor ligand activity | MF | 12 | 12/66 | 486/18337 | 1.41E-07 | 7.65E-06 | 5.08E-06 |
| GO:0030546 | Signaling receptor activator activity | MF | 12 | 12/66 | 492/18337 | 1.61E-07 | 7.65E-06 | 5.08E-06 |
| GO:0140375 | Immune receptor activity | MF | 7 | 7/66 | 136/18337 | 5.72E-07 | 2.27E-05 | 1.50E-05 |
| GO:0001664 | G protein-coupled receptor binding | MF | 9 | 9/66 | 289/18337 | 8.93E-07 | 3.04E-05 | 2.01E-05 |
| GO:0046982 | Protein heterodimerization activity | MF | 9 | 9/66 | 324/18337 | 2.30E-06 | 6.83E-05 | 4.53E-05 |
| GO:0001530 | Lipopolysaccharide binding | MF | 4 | 4/66 | 32/18337 | 5.10E-06 | 0.000124922 | 8.29E-05 |
| GO:0071723 | Lipopeptide binding | MF | 3 | 3/66 | 10/18337 | 5.25E-06 | 0.000124922 | 8.29E-05 |

**Table S7.** Classification of KEGG pathways enriched in hub genes.

| KEGG classification | Relative abundance |
| --- | --- |
| Signal transduction | 0.126315789 |
| Immune system | 0.105263158 |
| Cancer: specific types | 0.089473684 |
| Endocrine system | 0.084210526 |
| Infectious disease: viral | 0.057894737 |
| Infectious disease: bacterial | 0.052631579 |
| Cell growth and death | 0.042105263 |
| Immune disease | 0.042105263 |
| Cancer: overview | 0.036842105 |
| Carbohydrate metabolism | 0.031578947 |
| Digestive system | 0.031578947 |
| Neurodegenerative disease | 0.031578947 |
| Endocrine and metabolic disease | 0.031578947 |
| Infectious disease: parasitic | 0.031578947 |
| Cellular community - eukaryotes | 0.026315789 |
| Cardiovascular disease | 0.026315789 |
| Signaling molecules and interaction | 0.021052632 |
| Drug resistance | 0.021052632 |
| Transport and catabolism | 0.015789474 |
| folding sorting and degradation | 0.010526316 |
| Aging | 0.010526316 |
| Criculatory system | 0.010526316 |
| Excretory system | 0.010526316 |
| Nervous system | 0.010526316 |
| Development and regeneration | 0.010526316 |
| Substance dependence | 0.010526316 |
| Nucleotide metabolism | 0.005263158 |
| Cell motility | 0.005263158 |
| Sensory system | 0.005263158 |
| Environmental adaptation | 0.005263158 |

**Table S8.** KEGG pathways in signal transduction classification.

| ID | Description | Count | pvalue | GeneRatio | BgRatio | p.adjust | qvalue |
| --- | --- | --- | --- | --- | --- | --- | --- |
| hsa04064 | NF-kappa B signaling pathway | 11 | 4.33158E-10 | 11/65 | 104/8146 | 5.54984E-09 | 1.88082E-09 |
| hsa04022 | cGMP-PKG signaling pathway | 11 | 6.64423E-08 | 11/65 | 167/8146 | 5.44827E-07 | 1.8464E-07 |
| hsa04668 | TNF signaling pathway | 10 | 1.52926E-08 | 10/65 | 112/8146 | 1.42499E-07 | 4.82924E-08 |
| hsa04066 | HIF-1 signaling pathway | 9 | 1.68727E-07 | 9/65 | 109/8146 | 1.15297E-06 | 3.90737E-07 |
| hsa04020 | Calcium signaling pathway | 9 | 0.000109072 | 9/65 | 240/8146 | 0.00041407 | 0.000140327 |
| hsa04010 | MAPK signaling pathway | 9 | 0.000496634 | 9/65 | 294/8146 | 0.001497206 | 0.000507397 |
| hsa04151 | PI3K-Akt signaling pathway | 9 | 0.001851148 | 9/65 | 354/8146 | 0.004865196 | 0.001648796 |
| hsa04068 | FoxO signaling pathway | 8 | 8.43989E-06 | 8/65 | 131/8146 | 4.11947E-05 | 1.39607E-05 |
| hsa04015 | Rap1 signaling pathway | 8 | 0.000243718 | 8/65 | 210/8146 | 0.000819052 | 0.000277573 |
| hsa04630 | JAK-STAT signaling pathway | 7 | 0.000285538 | 7/65 | 162/8146 | 0.000929133 | 0.000314879 |
| hsa04371 | Apelin signaling pathway | 6 | 0.000800745 | 6/65 | 139/8146 | 0.002379025 | 0.000806242 |
| hsa04024 | cAMP signaling pathway | 6 | 0.008114052 | 6/65 | 221/8146 | 0.015401674 | 0.005219566 |
| hsa04071 | Sphingolipid signaling pathway | 5 | 0.002511742 | 5/65 | 119/8146 | 0.006129847 | 0.002077381 |
| hsa04072 | Phospholipase D signaling pathway | 5 | 0.00637069 | 5/65 | 148/8146 | 0.013191834 | 0.00447066 |
| hsa04350 | TGF-beta signaling pathway | 4 | 0.006581475 | 4/65 | 94/8146 | 0.013358439 | 0.004527122 |
| hsa04370 | VEGF signaling pathway | 3 | 0.011471642 | 3/65 | 59/8146 | 0.021186365 | 0.007179975 |
| hsa04012 | ErbB signaling pathway | 3 | 0.030126472 | 3/65 | 85/8146 | 0.047875401 | 0.016224783 |
